# Characterization of the SARS-CoV-2 ExoN (nsp14^ExoN^–nsp10) complex: implications for its role in viral genome stability and inhibitor identification

**DOI:** 10.1093/nar/gkab1303

**Published:** 2022-01-17

**Authors:** Hannah T Baddock, Sanja Brolih, Yuliana Yosaatmadja, Malitha Ratnaweera, Marcin Bielinski, Lonnie P Swift, Abimael Cruz-Migoni, Haitian Fan, Jeremy R Keown, Alexander P Walker, Garrett M Morris, Jonathan M Grimes, Ervin Fodor, Christopher J Schofield, Opher Gileadi, Peter J McHugh

**Affiliations:** Department of Oncology, MRC Weatherall Institute of Molecular Medicine, University of Oxford, John Radcliffe Hospital, Oxford OX3 9DS, UK; Department of Oncology, MRC Weatherall Institute of Molecular Medicine, University of Oxford, John Radcliffe Hospital, Oxford OX3 9DS, UK; Centre for Medicines Discovery, University of Oxford, Old Road Campus Research Building, Roosevelt Drive, Oxford OX3 7DQ, UK; Department of Oncology, MRC Weatherall Institute of Molecular Medicine, University of Oxford, John Radcliffe Hospital, Oxford OX3 9DS, UK; Chemistry Research Laboratory, Department of Chemistry and the Ineos Oxford Institute for Antimicrobial Research, University of Oxford, Mansfield Road, Oxford OX1 3TA, UK; Department of Oncology, MRC Weatherall Institute of Molecular Medicine, University of Oxford, John Radcliffe Hospital, Oxford OX3 9DS, UK; Department of Oncology, MRC Weatherall Institute of Molecular Medicine, University of Oxford, John Radcliffe Hospital, Oxford OX3 9DS, UK; Sir William Dunn School of Pathology, University of Oxford, South Parks Road, Oxford OX1 3RE, UK; Division of Structural Biology, Henry Wellcome Building for Genomic Medicine, University of Oxford, Oxford OX3 7BN, UK; Sir William Dunn School of Pathology, University of Oxford, South Parks Road, Oxford OX1 3RE, UK; Department of Statistics, University of Oxford, 24-29 St Giles', Oxford OX1 3LB, UK; Division of Structural Biology, Henry Wellcome Building for Genomic Medicine, University of Oxford, Oxford OX3 7BN, UK; Diamond Light Source Ltd, Harwell Science & Innovation Campus, Didcot OX11 0DE, UK; Sir William Dunn School of Pathology, University of Oxford, South Parks Road, Oxford OX1 3RE, UK; Chemistry Research Laboratory, Department of Chemistry and the Ineos Oxford Institute for Antimicrobial Research, University of Oxford, Mansfield Road, Oxford OX1 3TA, UK; Centre for Medicines Discovery, University of Oxford, Old Road Campus Research Building, Roosevelt Drive, Oxford OX3 7DQ, UK; Department of Oncology, MRC Weatherall Institute of Molecular Medicine, University of Oxford, John Radcliffe Hospital, Oxford OX3 9DS, UK

## Abstract

The SARS-CoV-2 coronavirus is the causal agent of the current global pandemic. SARS-CoV-2 belongs to an order, *Nidovirale*s, with very large RNA genomes. It is proposed that the fidelity of coronavirus (CoV) genome replication is aided by an RNA nuclease complex, comprising the non-structural proteins 14 and 10 (nsp14–nsp10), an attractive target for antiviral inhibition. Our results validate reports that the SARS-CoV-2 nsp14–nsp10 complex has RNase activity. Detailed functional characterization reveals nsp14–nsp10 is a versatile nuclease capable of digesting a wide variety of RNA structures, including those with a blocked 3′-terminus. Consistent with a role in maintaining viral genome integrity during replication, we find that nsp14–nsp10 activity is enhanced by the viral RNA-dependent RNA polymerase complex (RdRp) consisting of nsp12–nsp7–nsp8 (nsp12–7–8) and demonstrate that this stimulation is mediated by nsp8. We propose that the role of nsp14–nsp10 in maintaining replication fidelity goes beyond classical proofreading by purging the nascent replicating RNA strand of a range of potentially replication-terminating aberrations. Using our developed assays, we identify drug and drug-like molecules that inhibit nsp14–nsp10, including the known SARS-CoV-2 major protease (M^pro^) inhibitor ebselen and the HIV integrase inhibitor raltegravir, revealing the potential for multifunctional inhibitors in COVID-19 treatment.

## INTRODUCTION

From late 2019 and throughout 2020, the SARS-CoV-2 virus, which causes the disease COVID-19, has spread across the globe, infecting upwards of two-hundred million people to date and killing over five million of these (coronavirus.jhu.edu). A detailed understanding of the mechanistic aspects of the SARS-CoV-2 life and infectivity cycles are urgently required as are drugs that curb its replication and virulence.

SARS-CoV-2 is a coronavirus (CoV), of the *Coronaviridae* family in the *Nidovirales* order. One characteristic of these CoVs is their relatively large single-stranded RNA genomes, i.e. ∼30 kb in the case of SARS-CoV-2 (1). Perhaps necessarily, CoVs typically have a replication fidelity rate of an order of 10^−6^ to 10^−7^, several orders of magnitude more accurate than that of most RNA viruses (typically ∼10^−3^ to 10^−5^) (2). To maintain the fidelity of these genomes during replication, CoVs rely on a complex of two non-structural proteins, nsp14 (also known as ExoN) and nsp10 (2–4). The importance of this enhanced level of replication fidelity has been demonstrated in studies that disrupt or inactivate the activity of nsp14–nsp10, where reduced virulence and pathogenesis is seen in mouse and cellular models (4–6). Therefore, targeting nsp14–nsp10 is an attractive therapeutic strategy, either as a standalone option, or as an adjuvant to other agents that target other features of the viral replication cycle (7).

The nsp14–nsp10 proteins form a complex where a ribonuclease activity is conferred by the DEDD catalytic motif of nsp14, but where nsp10 plays a key role in conferring full activity (3,8,9). Studies of the closely related SARS-CoV complex, that caused the 2003 SARS epidemic, identified 3′-5′ exonuclease activity. Based on the ability of nsp14–nsp10 to excise terminally mismatched ribonucleotides, one function ascribed to the complex (by analogy with replicative DNA polymerases) is a ′proofreading′ activity (3,10), although other roles in viral replication have been proposed (11). Consistent with a role of this proofreading in maintaining genome stability, nuclease-inactivating mutations in CoV DEDD motifs cause an elevated level of replication errors, impaired replication, and in some cases lethal mutagenesis (4,5,12). Moreover, nsp14 interacts with the CoV RNA-dependent RNA polymerase (RdRp), i.e. the nsp12 subunit of the nsp12–nsp7–nsp8 (nsp12–7–8) complex, integrating the nuclease activities of nsp14 with the replicative process, although the molecular details of this interaction remain only partly characterised (10,11). The nsp14 protein also contains a functionally distinct (from its nuclease activity) *S*-adenosyl methionine (SAM)-dependent RNA guanine-N7 methyl transferase (MTase) activity (13–15). This activity is involved in the third step of production of the mature 5′-RNA CoV cap structure (cap-1), by methylating a 5′–5′ triphosphate GpppN generating the cap-0 intermediate.

Here, we describe purification of the SARS-CoV-2 nsp14–nsp10 complex and present detailed a biochemical characterization of its nuclease activity and substrate profile. We find that the complex is a highly versatile nuclease, which not only has the potential to act in a proofreading capacity, but which is also capable of processing a structurally diverse range of RNA molecules, in both ssRNA and dsRNA. Notably, some of the activities observed do not require a free 3′-terminus, implying that nsp14–nsp10 has both exo- and endonuclease-like activities. We propose that nsp14–nsp10 may act broadly to remove structures accumulating within the nascent replicating RNA strand that would potentially affect high fidelity extension by the SARS-CoV-2 RNA-dependent RNA polymerase (RdRp) (16). The nuclease activity of nsp14–nsp10 is enhanced by the viral RdRp; we demonstrate that the nsp8 subunit of the RdRp complex (nsp12–nsp7–nsp8), specifically enhances the nuclease activity of nsp14–nsp10. This may provide some insight as to the relationship between the polymerase and nuclease complexes *in vivo*. We also used the assay systems we developed to screen for inhibitors, identifying several drugs and drug-like molecules that might be developed or repurposed to inhibit the nuclease activity of the nsp14–nsp10 complex.

## MATERIALS AND METHODS

### Expression and purification of wildtype and nuclease inactive (nsp14^D113A/E115A^) nsp14–nsp10 complexes

The N-terminally His-tagged nsp14–nsp10 protein complex was produced using a ‘bi-cistronic’ vector in which DNA encoding for nsp14 is followed by that for a ribosome binding site and the untagged nsp10 ORF. Vectors and sequences can be found at: https://www.addgene.org/159613/. The recombinant protein was generated by transformation into *Escherichia coli* BL21 Rosetta2 (DE3) cells and expression in Terrific Broth media supplemented with 30 μg ml^−1^ kanamycin and 10 mM ZnCl_2_. The culture was incubated with shaking (200 rpm) at 37°C until an OD_600_ of 2.0 was reached. Cultures were then transferred to an incubator at 18°C for 30 min before induction with 1 mM isopropyl β-d-1-thiogalactopyranoside (IPTG) and incubated for 18 hours with shaking (200 rpm).

Cells were harvested by centrifugation at 6000 × g for 30 min at 4°C. The cell pellet was resuspended in lysis buffer (50 mM Tris–HCl pH 8.0, 500 mM NaCl, 10 mM imidazole, 5% v/v glycerol and 1 mM tris(2-carboxyethyl) phosphine (TCEP)) and lysed by sonication. The lysate was clarified by centrifugation at 40 000 × g for 30 min, and the supernatant was loaded onto an equilibrated (lysis buffer) immobilised metal affinity chromatography column (IMAC) (Ni-IDA Sepharose, GE Healthcare).

The immobilized protein was washed with lysis buffer and eluted with elution buffer (50 mM Tris–HCl pH 8.0, 500 mM NaCl, 300 mM imidazole, 5% v/v glycerol and 1 mM TCEP). The protein-containing fractions were pooled and dialysed overnight at 4°C in dialysis buffer (25 mM Tris–HCl pH 8.0, 150 mM NaCl, 5% v/v glycerol and 1 mM TCEP) supplemented with recombinant tobacco etch virus (rTEV) protease for cleavage of the N-terminal 6xHis tag. The protein was then subjected to a second IMAC step to remove His-tagged rTEV protease, cleaved His tag, and uncleaved His-tagged nsp14–nsp10 complex.

The cleaved nsp14–nsp10 complex was concentrated to 1 ml using a 50 kDa MWCO centrifugal concentrator. The protein was then further purified by size exclusion chromatography (SEC) using a Superdex 200 Increase 10/300 GL column equilibrated with SEC buffer (25 mM HEPES pH 7.5, 150 mM NaCl, 5% v/v glycerol, 2 mM TCEP) at 0.8 ml/min. The purified protein was concentrated to 0.5 mg ml^−1^ and was snap frozen in liquid nitrogen for later use.

Monomeric N-terminally His-tagged nsp14 protein was produced by expression in Terrific Broth supplemented with 30 μg ml^–1^ kanamycin and 10 mM ZnCl_2_ from the same vector. The 5 ml of overnight culture were transferred into 500 ml of media and incubated at 37°C with shaking (200 rpm) until an OD_600_ of 2.0 was reached. Cultures were then transferred to an incubator at 18°C for 16–18 h.

Protein concentrations were determined using a NanoDrop™ ONE^c^ machine (Thermo Scientific). The presence of the nsp14–nsp10 complex was confirmed *via* SDS-PAGE and ESI-Q-TOF (electrospray-ionization quadrupole time-of-flight) mass spectroscopy (MS).

### Purification of wildtype and mutant nsp12, nsp7 and nsp8

Nsp12, nsp7, and nsp8 were purified as reported (17). Briefly, full-length nsp12, nsp7 and nsp8 (codon optimized for insect cell expression) were cloned and purified from *Sf9* insect cells (nsp12), or BL21 *E. coli* (nsp 7, nsp8). Nsp12 was purified by an initial IgG Sepharose chromatography step, followed by overnight TEV protease cleavage, and subsequent SEC (Superdex 200, Increase 10/300 GL column; GE Healthcare). Nsp7 and 8 were purified by Glutathione-Sepharose (GE Healthcare), followed by PreScission protease cleavage, and subsequently passed through a Superdex 75 (Increase 10/300 GL column; GE Healthcare) column. The identity and purity of purified proteins was validated by SDS-PAGE (Supplementary Figure 3A).

### Generation of 5′ radiolabelled substrates

10 pmol of single-stranded (ss) RNA or DNA (Eurofins MWG Operon, Germany) was incubated with 6.8 pmol γ-^32^P-ATP (Perkin Elmer), and 10 U T4 PNK (ThermoFisher Scientific) at 37°C for 1 h. This solution was passed through a P6 Micro Bio- Spin chromatography column (BioRad) to remove unincorporated label and diluted accordingly in nuclease-free ultrapure H_2_O.

For double-stranded (ds) structures, radiolabelled RNA or DNA was annealed to unlabelled complementary molecules at a 1:1.5 ratio to give a final concentration of 100 nM in 10 mM Tris–HCl (pH 7.5), 50 mM NaCl, and 1 mM EDTA by heating the sample to 95°C for 3 min before cooling to room temperature. A detailed list of all oligonucleotide sequences is in Supplementary Table 1.

### Generation of a single nucleotide RNA ladder

10 pmol of 5′ radiolabelled ssRNA was incubated in 50 mM potassium acetate (pH 7.9), 20 mM Tris-acetate (pH 7.9), 10 mM magnesium acetate (pH 7.9) and 1 mM DTT for 2, 5, 10 and 15 min at 95°C. 5 μl of stop solution (95% formamide, 10 mM EDTA, 0.25% xylene cyanol, 0.25% bromophenol blue) was then added to each sample; samples were combined and analysed using a 20% denaturing polyacrylamide gel (40% solution of 19:1 acrylamide:bis-acrylamide, BioRad and 7 M urea (Sigma Aldrich)) in 1× TBE (Tris–borate–EDTA) buffer. Electrophoresis was carried out at 525 V for 1.5 h; gels were subsequently fixed for 60 min in fixing solution (50% methanol, 10% acetic acid), and dried at 80°C for 2 h under a vacuum. The dried gels were exposed to a Kodak phosphorimager screen and scanned using a Typhoon 9400 instrument (GE).

### Nuclease assays

Nuclease assays were carried out in a 10 μl final volume containing 25 mM HEPES–KOH, pH 7.5, 50 mM NaCl, 5 mM MgCl_2_, 5% glycerol, 1.0 mM DTT, 10 nM RNA or DNA substrate and serial two-fold dilutions from 500 nM of nsp14–nsp10 complex or nsp14. Reactions were incubated at 37°C for 45 min, and quenched by the addition of 5 μl stop solution (95% formamide (v/v), 10 mM EDTA, 0.25% xylene cyanol, 0.25% bromophenol blue) and boiling at 95 °C for 3 min.

Reactions were analysed by 20% denaturing polyacrylamide gel electrophoresis (40% solution of 19:1 acrylamide:bis-acrylamide, BioRad) and 7 M urea (Sigma Aldrich)) in 1× TBE (Tris–borate–EDTA) buffer. Electrophoresis was carried out at 525 V for 1.5 h; gels were subsequently fixed for 60 min in fixing solution (50% methanol, 10% acetic acid), and dried at 80°C for 2 h under a vacuum. The dried gels were exposed to a Kodak phosphorimager screen and scanned using a Typhoon 9400 instrument (GE).

Nuclease assays with the addition of the nsp12–7–8 complex (as indicated) were carried out as above, with the following modifications. To facilitate complex formation, 200 nM of nsp12, 600 nM nsp7 and 600 nM of nsp8 (a molar ratio of 1:3:3) were incubated with the relevant RNA substrate (10 nM) for 5 min at 37°C, before the nuclease reaction was started by addition of nsp14–nsp10. Nsp14–nsp10 final concentrations (unless otherwise indicated) were 100 nM.

All gel-based assays were repeated a minimum of three times and across a minimum of three preparations of nsp14–nsp10. Where applicable, gel-based nuclease assays were quantified by analysing the product formation (digestion) of the radiolabelled RNA substrate(s). Gel images collected on a Typhoon scanner were analysed in ImageJ to determine the amount of substrate running at the top of the gel (undigested) in comparison to the amount of substrate that ran further into the gel (product) reported as percent digested. This was plotted versus protein concentration to indicate digestion rate. Error is standard error. Three or more gels were analysed for each substrate.

### Nuclease inhibition assays

Inhibitor exonuclease assays were carried out in 10 μl volumes containing 25 mM HEPES–KOH, pH 7.5, 50 mM NaCl, 5 mM MgCl_2_, 5% glycerol, 1.0 mM DTT and 100 nM nsp14–nsp10. Inhibitors were dissolved in DMSO (100 μM) and serially diluted 2-fold from the 100 μM stock solution; the DMSO concentration was kept constant at 0.1% (v/v) in the final reaction mixture. Inhibitors were incubated for 10 min at room temperature with the nsp14–nsp10 complex; reactions were initiated by the addition of RNA substrate (10 nM), incubated at 37°C for 45 min, then quenched by the addition of 5 μl stop solution (95% formamide, 10 mM EDTA, 0.25% xylene cyanol, 0.25% bromophenol blue) and boiled at 95°C for 3 min. Reactions were analysed by 20% denaturing PAGE, fixed, dried, then imaged as described above.

### Methyltransferase assays

The methyltransferase activity of nsp14–nsp10 was assayed by detection of released SAH by using the EPIgeneous™ methyltransferase kit (CisBio Bioassays) according to the manufacturer's instructions. The methyltransferase reaction was conducted at room temperature in an 8 μl reaction volume with 10 nM nsp14–nsp10, 5 μM Ultrapure SAM (CisBio), 0.14 mM GP_3_G RNA cap analogue (Jena Bioscience) in a reaction buffer consisting of HEPES–KOH pH 7.6, 150 mM NaCl and 0.5 mM DTT for 20 min and quenched by the addition of 2 μl 5 M NaCl. Next, 2 μl of Detection Buffer 1 (CisBio) was added to the reaction. After 10 minutes, 4 μl of 16x SAH-d2 conjugate solution (CisBio) was added. After 5 min, 4 μl of 1× α-SAH Tb Cryptate antibody solution was added to the reaction mixture. Homogenous Time Resolved Fluorescence (HTRF) measurements were taken after 1 h on a SpectraMax M3e (Molecular Devices) plate reader. Readings were taken at emission wavelengths of λ = 665 nm and λ = 620 nm after excitation at λ = 337 nm. The experimental HTRF ratio (HTRF_exp_) was then calculated as ratio of emission intensities: λ = 665/λ = 620. To reach the normalised HTRF ratio, HTRF ratio measurements were also taken of wells without enzyme (*E*_0_) and without SAH-d2 (*d*2_0_), representing the maximum and minimum achievable HTRF values, respectively. The normalised HTRF ratio was then calculated as a linear transformation of the experimental HTRF ratio, the *E*_0_ ratio, and the *d*2_0_ ratio: $\;{\rm HTRF}\; =$$\frac{{{{\rm HTRF}_{{\rm exp}}} - d{2_0}}}{{{E_0} - d{2_0}}}$.

### Molecular docking

The structure of the best available SARS-CoV nsp14–nsp10 entry in the Protein Data Bank (PDB), PDB ID: 5NFY (3.38 Å resolution) (10), was overlaid with another SARS-CoV nsp14–nsp10 structure (PDB: 5C8U, resolution 3.40 Å^8^) to add the missing active site Mg^2+^ in PDB: 5NFY from that in 5C8U. The coordinates were then translated such that the centre of mass was closer to the origin, (0,0,0), and prepared for docking with AutoDockTools. Blind docking was carried out using AutoDock Vina using a grid box of 64 Å × 70 Å × 126 Å centred on (–0.476, 5.300, 8.298), encompassing the entire protein surface. A second set of more focused dockings was performed with AutoDock Vina focused on the active site, with the grid box specified to be 30 Å × 24 Å × 24 Å centred on (–5.196, –9.373, –7.837). Predicted AutoDock Vina affinities of each docked binding mode for each ligand were extracted, along with the distance from the centre of the binding mode to the catalytic Mg^2+^, and to the centre of mass of GpppA in the MTase binding site. Docked poses were analysed using PyMOL and rendered with ChimeraX.

## RESULTS

### SARS-CoV-2 nsp14–nsp10 is an RNA processing nuclease

**Figure 1.**
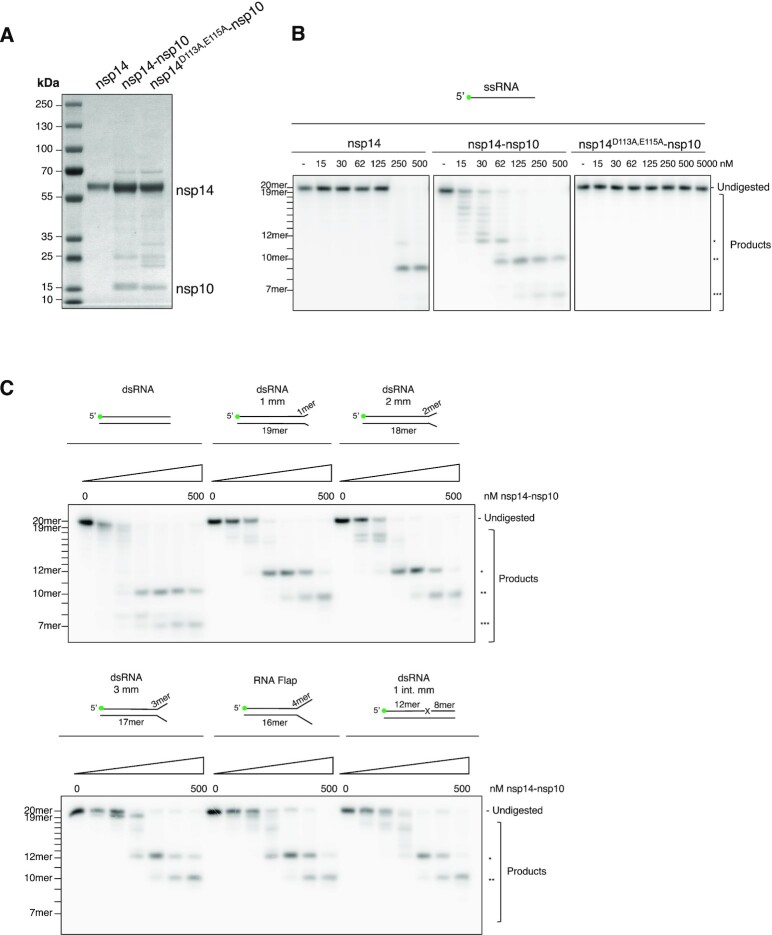
SARS-CoV-2 nsp14–nsp10 is a versatile RNA proofreading nuclease.(**A**) SDS-PAGE of purified nsp14 alone and nsp14–nsp10 and an inactive control complex bearing substitutions at residues D113 and E115 (nsp14^D113A,E115A^–nsp10) showing the purity of the purified proteins. Predicted molecular weights are nsp14: 60 034 Da (wild-type nsp14–nsp10), 59 931 Da (nsp14^D113A,E115A^–nsp10) and nsp10: 14 790 Da. (**B**) Nsp14–nsp10 is an RNase able to digest a 20-mer ssRNA oligo (Oligo 2 in Supplementary Table 1A) in a single-nucleotide fashion from the 3'-end terminating at the eighth ribonucleotide from the 3'-end (labelled *) and further incising closer to the 5'-end to generate 10-mer and 7-mer products (labelled ** and *** respectively). Nsp14 alone is able to generate the 12-mer and 10-mer products at significantly higher protein concentrations. The predicted inactive nsp14^D113A,E115A^–nsp10 complex exhibits no discernible activity, even at ten-fold higher concentrations compared with the wild-type complex. (**C**) Nsp14–nsp10 is an exo- and endo-nuclease acting on a variety of RNA substrates, including RNA substrates with mismatched termini and flaps with no preference for mismatched ribonucleotides. Quantification in Suppl. Figure 2B. mm: mismatch. int. mm: internal mismatch. Increasing concentrations of protein (as indicated) were incubated with substrate (37°C, 45 min). Reactions were analysed by 20% denaturing PAGE. Size of products was determined as shown in Supplementary Figure 1E. Main products are labelled *, ** and *** corresponding to 12-mer, 10-mer and 7-mer respectively. All oligonuleotides used are given in Supplementary Table 1A and B.

Employing codon optimised constructs expressed in *E. coli*, we purified the nsp14–nsp10 complex to near homogeneity (Figure 1A). Bands between the nsp14 and nsp10 proteins visible by SDS-PAGE were shown to be nsp14 degradation products by trypsin digestion followed by fragmentation mass spectrometry. The nsp14–nsp10 complex eluted from size exclusion chromatography (SEC) in a volume consistent with a dimer of monomers (ab complex, 65 kDa) (Supplementary Figure 1A); the identity of the proteins was confirmed by intact mass spectrometry (Supplementary Figure 1B). In addition to the wild-type complex, we also purified a control complex bearing substitutions at residues nsp14 D113 and nsp14 E115 (nsp14^D113A,E115A^–nsp10) which, by comparison with the analogous SARS-CoV complex, is expected to be catalytically inactive (Figure 1A) (8). We also purified the nsp14 (ExoN) subunit alone to determine its activity; note: purified recombinant nsp10 produced in bacteria did not respond well to iterative freeze-thaw cycles, consistent with its association with nsp14 helping to maintain solubility, as previously proposed (8) (Figure 1A).

To investigate whether SARS-CoV-2 nsp14–nsp10 acts as an RNA nuclease, analogous to the SARS-CoV complex, we utilised a 20-mer ssRNA substrate radiolabelled at its 5′-terminus (8). Initially, we tested the activity of nsp14 alone and, consistent with its predicted role as a nuclease, we observed nucleolytic digestion, albeit at nsp14 concentrations >250 nM employing 10 nM substrate (Figure 1B).

It is reported that whilst isolated SARS-CoV nsp14 is active as a nuclease, its activity is substantially enhanced by association with nsp10 (3,8). Indeed, the results of incubating the ssRNA substrate with SARS-CoV-2 nsp14–nsp10 confirmed that nsp10 substantially enhances nsp14 activity, with efficient digestion by nsp14–nsp10 being observed at the lowest concentration of the complex employed (15 nM nsp14–nsp10; 10 nM substrate) (Figure 1B). Time-course assays containing 30 nM nsp14–nsp10 for ssRNA and 60 nM nsp14–nsp10 for dsRNA were used to define the linear range of product formation (Supplementary Figure 1C). Based on the results, we comprehensively explored activity and substrate selectivity employing concentrations of 15–500 nM nsp14–nsp10 for a 45-minute incubation period (Supplementary Figure 1C).

To test that the observed nuclease activity is intrinsic to the nsp14–nsp10 complex, we employed two methods. Firstly, we purified the predicted nuclease inactive complex nsp14^D113A,E115A^–nsp10 and assayed its activity with the 20-mer substrate. No activity was observed, even at concentrations 10-fold higher than the highest concentration employed with the wild-type complex (5000 nM for nsp14^D113A,E115A^–nsp10 versus 500 nM for nsp14–nsp10; Figure 1B). Secondly, we purified the wild-type nsp14–nsp10 complex by SEC and assayed the eluted fractions for RNase activity. The elution profile of the nsp14–nsp10 complex SEC coincided with the peak for RNase activity (Supplementary Figure 1F), providing further evidence that the observed activities are intrinsic to nsp14–nsp10 and not due to a contaminant.

We next examined the activity of nsp14–nsp10 on sequence-related ssRNA substrates of varying length. Whilst no activity was observed with a 10-mer substrate, possibly because it is too short to permit catalytically productive binding to nsp14–nsp10, robust activity was observed with the 20-mer and 30-mer substrates, with a preference for the 20-mer substrate (Supplementary Figure 1D). For both 20-mer and 30-mer substrates, a complex digestion pattern was observed (Supplementary Figure 1D). We observed RNA laddering close to the 3′-terminus, consistent with the previously reported 3′-exonuclease activity of nsp14–nsp10 (9), but also several additional prominent bands representing cleavage further from the 3′-end. We investigated the precise size of the major products released by the nsp14–nsp10 on the 20-mer substrate, by performing limited hydrolysis on the 20-mer substrate to provide a single-nucleotide ‘molecular weight marker’ (Supplementary Figure 1E). This enabled us to determine that the laddering products observed at the top of the gel correspond to fragments digested in a single-nucleotide fashion from the 3′-end, and that this processing terminates at the 8th ribonucleotide from the 3′-end (labelled with *). Two additional prominent bands were identified, corresponding to cleavage at the 10th and 13th ribonucleotides from the 3′-end, releasing two 10-mer and 7-mer products (labelled on Figure 1B and Supplementary Figure 1E as ** and ***, respectively).

As previous biochemical and structural studies imply that the nuclease activity of the ExoN family of nucleases is dependent upon divalent metal cations, we sought evidence that this is the case for SARS-CoV-2 nsp14–nsp10 (3,8,9). First, we determined which divalent cations support maximal activity of the complex. Both magnesium and manganese promoted the RNase activity of nsp14–nsp10, whereas zinc was inhibitory (Supplementary Figure 1G). Consistent with a requirement for metal ions for activity, three metal-chelating agents, ethylenediaminetetraacetic acid (EDTA), ethylene glycol-bis (*b*-aminoethyl ether)-*N*,*N*,*N*’,*N*’-tetraacetic acid (EGTA), and *o*-phenanthroline were inhibitory, with a particular sensitivity to EDTA (Supplementary Figure 1G). For further studies, we chose to perform reactions in the presence of magnesium as this efficiently supports nsp14–nsp10 activity while being highly abundant in the mammalian cytoplasm.

### SARS-CoV-2 nsp14–nsp10 can process dsRNA and dsRNA substrates containing terminal mismatches

It has been reported that the SARS-CoV nsp14–nsp10 complex degrades dsRNA substrates and substrates containing mismatches of up to 4-ribonucleotides at their 3′-termini (3,9). To address this possibility for the SARS-CoV-2 complex, we generated substrates containing 1-, 2-, 3- or 4-ribonucleotide mismatches at their 3′-termini, where the 4-ribonucleotide mismatch effectively introduces a small 3′-flap allowing us to examine the impact of this secondary structure on activity. Standard duplex RNA was used for comparison. We observed digestion across all of these four mismatched structures (Figure 1C and quantified in Supplementary Figure 2B), although nucleolytic activity was reduced compared with standard dsRNA where a terminal mismatch is 2-nt or more. Interestingly, there were qualitative differences in the major products released, likely reflecting differential modes of association of the complex due to the structural variations in the substrates. We also examined a substrate containing a single mismatch near its centre (8-nucleotides from the 3′-end). This was also processed with an efficiency similar to the other substrates, exhibiting a qualitative pattern of digestion similar to the substrates containing the terminal mismatches (Figure 1C and quantified in Supplementary Figure 2B). Taken together, our data support the capacity of SARS-CoV-2 nsp14–nsp10 to process 3′ mismatched substrates and so potentially contributes to proofreading activity.

### Nsp14–nsp10 exhibits both 3′-exonuclease and a newly-identified endonucleolytic activity

Although we observe nuclease activity on mismatched RNA substrates for the SARS-CoV-2 nsp14–nsp10 complex, this does not appear to be preferential for mismatches with digestion occurring with no greater efficiency than for a simple duplex substrate (Figure 1C; quantified in Supplementary Figure 2B). Therefore, we performed an extensive evaluation of RNA structures, sequences, and modifications to better define the major activities of the complex to determine if the complex possesses previously unrealised activities.

**Figure 2.**
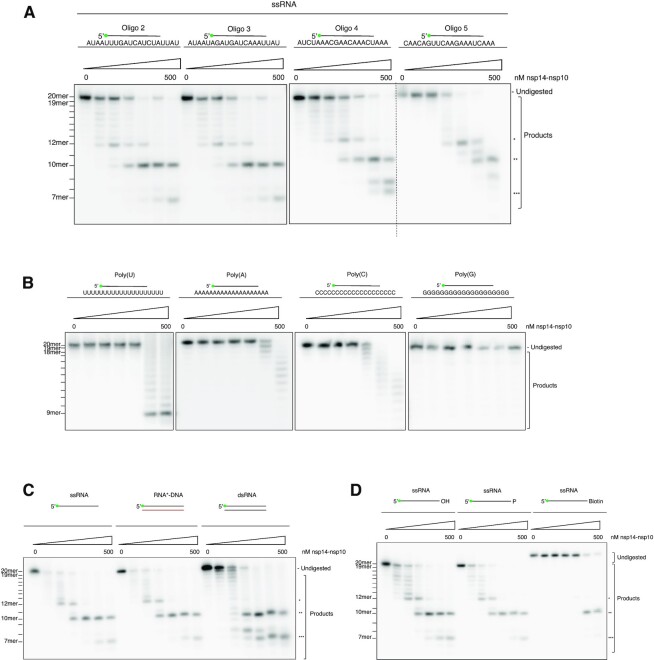
Nsp14–nsp10 exhibits both 3'-exonuclease activity and a newly-described endonucleolytic activity that extends beyond the classical role of a proofreading nuclease.(**A**) The nuclease activity of nsp14–nsp10 is not sequence specific on a mixed-sequence substrate. The complex shows indistinguishable digestion patterns on four 20-mer ssRNA substrates of different sequences and containing all four ribonucleotides. Oligonucleotides 2, 3, 4 and 5 were used respectively (see Supplementary Table 1A). (**B**) When presented with 20-mer Poly(U), Poly(A), Poly(C) and Poly(G) ssRNA (oligonucleotides 11, 12, 13 and 14 respectively, see Supplementary Table 1A), nsp14–nsp10 shows reduced and qualitatively altered activity, with a single nucleotide step-wise digestion from the 3'-end curtailing at the 9th–11th nucleotide from the 3'-end. (**C**) Nsp14–nsp10 processes ssRNA, the RNA strand of an RNA:DNA hybrid, and dsRNA with no preference for double-stranded substrates. For all structures, the labelled oligo is oligo 2 (see Supplementary Table 1A and B). (**D**) Nsp14–nsp10 has RNA exo- and endo- nuclease activities. With a substrate containing a 3'-biotin group, the characteristic exonucleolytic laddering of the substrate is lost and only endonucleolytic cleavage at positions furthest from the 3'-end is observed; substrates with a 3'-hydroxyl or phosphate exhibit nearly identical product formation profiles. Oligonucleotides 2, 15 and 16 were used respectively (see Supplementary Table 1A). Increasing concentrations of protein (as indicated) were incubated with substrate (37°C, 45 min); reactions were subsequently analysed by 20% denaturing PAGE. The size of products was determined as shown in Supplementary Figure 1E. Main products are labelled *, ** and *** corresponding to 12-mer, 10-mer and 7-mer respectively. All oligonucleotides used are indicated in Supplementary Table 1A and B.

To examine any RNA sequence dependency, we compared the activity of nsp14–nsp10 on the 20-mer substrate employed in Figure 1B with its activity on three additional sequence unrelated 20-mer ssRNAs, selected from the SARS-CoV-2 genome. All substrates were selected containing a mixed sequence, of all four bases, and have a negligible predicted capacity to form stable secondary structures under the reaction conditions used (see calculated minimum free energy values in Supplementary Table 1A). It is striking that the pattern and efficiency of digestion on these four substrates was virtually indistinguishable suggesting that, at least for RNA substrates of mixed sequence, the major determinant of digestion pattern is not sequence (Figure 2A).

However, when nsp14–nsp10 was presented with 20-mer poly(A), poly(U), poly(C) or poly(G) ssRNA substrates, the activity was substantially reduced and qualitatively altered (Figure 2B). For poly(U), step-wise digestion to the 11th ribonucleotide from the 3′-end was observed, whereas for the poly(A) substrate the pattern of digestion was similar, but was (at least predominantly) curtailed at the 8th or 9th nucleotide from the 3′-terminus. Digestion of the poly(C) substrate showed a similar pattern as observed for the poly(A) substrate, but activity was observed at one-fold lower enzyme concentration (125 nM nsp14–nsp10). Moreover, we did not observe evidence for digestion of the poly(G) substrate (Figure 2B). It is, nonetheless, important to note that the concentration of enzyme required to observe any digestion on the poly(U), poly(A) and poly(C) substrates (at least 125 nM nsp14–nsp10) is substantially higher than those required to efficiently digest RNA substrates of mixed sequence (compare Figures 2A and 2B).

To examine if the SARS-CoV-2 nsp14–nsp10 exhibits preference for ds- or ssRNA, we tested its activity on sequence-identical ssRNA, dsRNA and hybrid RNA:DNA substrates (where the RNA strand is 5′-radiolabelled) (Figure 2C). We did not observe a marked preference for either dsRNA or ssRNA (Figure 2C and quantified in Supplementary Figure 2C). Moreover, nsp14–nsp10 appears to be agnostic to the nature of the strand annealed to the RNA substrate strand, since the RNA component of the RNA:DNA hybrid was digested with efficiency equal to the ssRNA substrate (Figure 2C, and Supplementary Figure 2C).

Consistent with its role as an RNA nuclease, nsp14–nsp10 was unable to digest a ssDNA substrate, or the DNA strand of an RNA:DNA hybrid. Strikingly, on a ssDNA substrate containing an embedded ribonucleotide (analogous to the types of substrate processed by the ribonucleotide excision repair (RER) enzymes (18)), nsp14–nsp10 was able to nucleolytically incise the substrate adjacent to the ribonucleotide, whilst not processing the DNA portion (Supplementary Figure 2A).

To investigate whether any of the products observed in the assays described above could be attributed to endonuclease activity, we compared the activity of nsp14–nsp10 on three sequence-identical substrates bearing either a 3′-hydroxyl, 3′-phosphate, or 3′-biotin group (Figure 2D and Supplementary Figure 2D). Interestingly, the characteristic ladder of products extending from the full-length substrate (at the top of the gel) was largely abrogated when the 3′-hydroxyl was replaced by a biotin group only. However, the other major products (7-mer and 10-mer) observed on the substrate with the hydroxyl terminus were variably affected when the 3′-end was replaced with a phosphate or biotin. In the presence of the 3′-phosphate, the 10-mer product was still observed, being produced in approximately equal amounts as for the 3′-hydroxyl substrate, while the 7-mer product was observed at slightly higher concentrations (above 250 nM) compared to the 3′-hydroxyl substrate. With a 3′-biotin substrate, higher enzyme concentrations were required to observe any cleavage with predominantly the 10-mer product being observed at concentrations above 250 nM. The 12-mer product was still observed at concentrations above 125 nM, albeit faintly, whilst the 7-mer product was only observed at the highest concentration (500 nM). Together, these observations strongly suggest that SARS-CoV-2 not only has a 3′-exonuclease activity dependent upon the presence of an unblocked 3′-RNA terminus (leading to the laddering products we characteristically observe at the top of the gels), but also has endonucleolytic activity, cleaving at positions further from the 3′-end, generating a predominantly 10-mer product. These incisions occur independently of the presence of a 3′-hydroxyl or phosphate group, and therefore do not require engagement with a 3′-terminus prior to initial exonucleolytic processing at the 3′-terminus for their production.

To validate whether the 3′ biotin group effectively blocks the free 3′ end of the RNA substrate, we tested whether Exonuclease T can digest the biotin-modified substrate. Whilst Exonuclease T was (as expected) able to efficiently process both the 3′ phosphate and 3′ hydroxyl, its activity was clearly blocked by the presence of a 3′ biotin even at very high concentrations (4 units of Exonuclease T is ∼1 μM), suggesting that nsp14–nsp10 does indeed possess *bona fide* endoribonuclease activity (Supplementary Figure 2E).

Finally, we examined whether some common modifications of viral RNA impact on the RNase activity of nsp14–nsp10. We investigated the impact of 6-methyladenine, one of the most common modifications found in cytoplasmic mRNA (19), along with an artificial base modification, 2-methyladenine. Interestingly, the 6-methyladenine modification has been associated with viral evasion by the host innate immune system (19). We also examined the variant RNA base inosine, which can be generated by A-to-I editing and which is reported to enhance viral recognition by the innate immune sensors (20). When any of these modified bases were placed two ribonucleotides from the 3′-terminus of the 20-mer substrate, none affected the activity of nsp14–nsp10 when compared with an unmodified RNA substrate. Comparable results were obtained for Exonuclease T (Supplementary Figure 2F & Supplementary Figure 2G). We conclude that some common chemical modifications of RNA do not substantially affect nsp14–nsp10 RNase activity.

### The SARS-CoV-2 polymerase nsp12–7–8 complex enhances nsp14–nsp10 activity on a variety of RNA substrates

**Figure 3.**
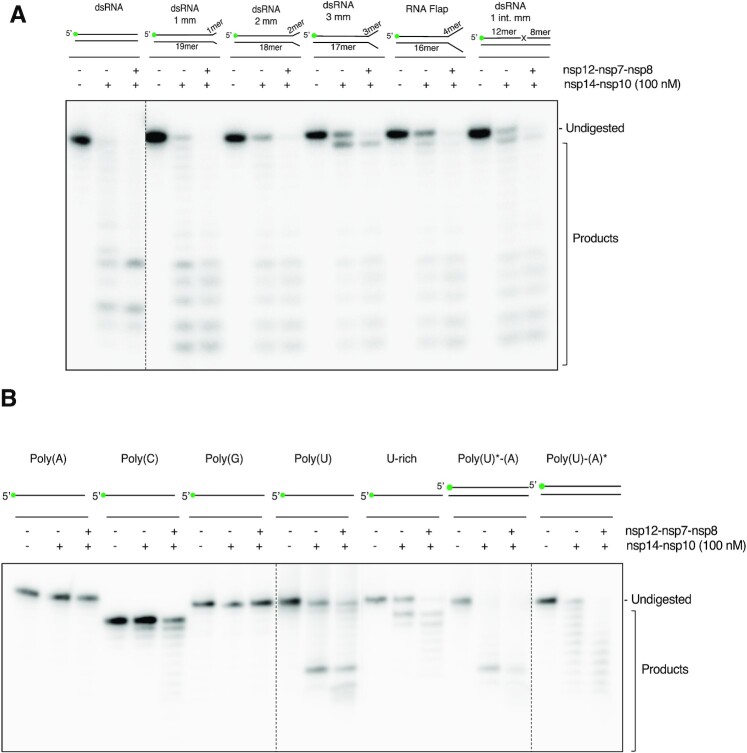
The SARS-CoV-2 polymerase nsp12-nsp7-nsp8 complex enhances nsp14–nsp10 nuclease activity on a variety of RNA substrates.(**A**) In the presence of the nsp12–7–8 polymerase complex, nsp14–nsp10 shows enhanced activity on a variety of RNA substrates, including RNA substrates with mismatched termini and flaps with no observed differential effects. mm: mismatch int mm: internal mismatch. B. When presented with 20-mer Poly(A), Poly(C), Poly(G), Poly(U) and U-rich ssRNA (oligonucleotides 12, 13, 14, 11 and 25 respectively, see Supplementary Table 1A) as well as double-stranded Poly(U)*-(A) and Poly(U)-(A)*, nsp14–nsp10 shows a more profound stimulation on repetitive sequences when nsp12–7–8 is present. Enhancement of nsp14–nsp10 nuclease activity by nsp12–7–8 is most pronounced on uracil-containing substrates. * Indicates labelled strand. The SARS-CoV-2 nsp12–7–8 polymerase complex (at a 1:3:3 molar ratio) was incubated with 10 nM substrate (37 °C, 5 min) prior to addition of 100 nM of nsp14–nsp10 at a 1:2 molar ratio of nsp14–nsp10 complex to nsp12–7–8 complex. Reactions were incubated at 37 °C for 45 min, then analysed by 20% denaturing PAGE. The size of products was determined as shown in Supplementary Figure 1E. Main products are labelled *, ** and *** corresponding to 12-mer, 10-mer and 7-mer respectively. Oligonucleotides used are given in Supplementary Table 1A and B.

An interaction between the RdRp complex (nsp12–7–8) and the nuclease complex (nsp14–nsp10) from SARS-CoV has been proposed, although the effects of this on the enzymatic activity of either complex have not been systematically elucidated (10,21,22). We examined the effect of adding the SARS-CoV-2 polymerase complex (nsp12–7–8) (Supplementary Figure 3A) on the nuclease activity of SARS-CoV-2 nsp14–nsp10. Given the putative proof-reading role of nsp14–nsp10, we tested the effect of adding nsp12–7–8 on both duplex RNA and a variety of mismatch containing substrates (Figure 3A). On all the tested substrates, the addition of nsp12–7–8 had a marked stimulatory effect on the nuclease activity of nsp14–nsp10, as evidenced by a decreased proportion of undigested substrate and an increase in lower molecular weight digestion products (compare lanes + and ++ in Figure 3A). We tested this effect on sequence diverse RNA substrates (Figure 3B). The results imply that repetitive sequences of any nature are largely inhibitory (Figures 2B and 3B). Addition of nsp12–7–8 still stimulated nsp14–nsp10 activity with some of these substrates (Figure 3B), but the effect was not uniform. On poly(A), poly(G), and poly(C) sequences nucleolytic digestion was negligible, both in the absence and presence of nsp12–7–8; however, on uracil containing substrates (namely the poly(U), and U-rich sequences) the stimulatory effect on nsp14–nsp10 activity was more pronounced, observed as a reduction in undigested substrate, and an increase in smaller digestion products (Figure 3B). This observation suggests that *in vivo* the presence of nsp12–7–8 may contribute to the substrate selectivity of nsp14–nsp10, as well as promoting its activity. As the greatest stimulatory effects were observed on a poly(U) and a U-rich substrate, we then tested duplex poly(A) and poly(U) substrates with either the poly(A) or poly(U) strand radiolabelled. On each of these substrates a stimulatory effect of nsp12–7–8 on nsp14–nsp10 was observed (Figure 3B).

**Figure 4.**
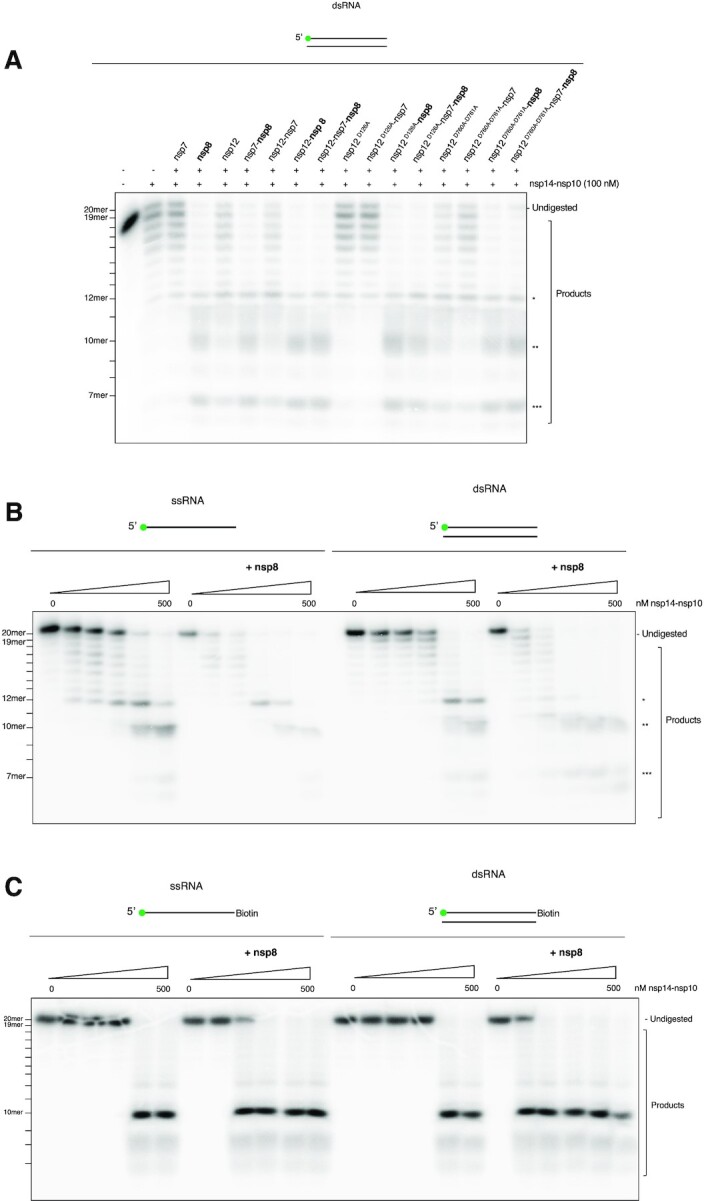
The SARS-CoV-2 polymerase nsp12–nsp7–nsp8 complex enhances nsp14–nsp10 nuclease activity on a variety of RNA substrates.(**A**) The SARS-CoV-2 primase, nsp8 enhances nsp14–nsp10 activity. The addition of each individual polymerase subunit, *i.e*.nsp12, nsp7, and/or nsp8, as well as different combinations reveal nsp8 as the major enhancer of nsp14–nsp10 activity. Mutant nsp12^D126A^ and nsp12^D760A-D761A^ show no substantial stimulation of activity of nsp14–nsp10 compared to nsp8 suggesting stimulation of nuclease activity is uncoupled from polymerase activity. (**B**) The presence of nsp8 stimulates the exonuclease activity of SARS-CoV-2 nsp14–nsp10. Increasing concentrations of nsp14–nsp10 were incubated with ss- or dsRNA (Oligo 2 and 3, see Supplementary Table 1A) and a fixed concentration of 300 nM nsp8 (37°C, 45 min). Reactions were analysed by 20% denaturing PAGE. (**C**) The presence of nsp8 stimulates the endonuclease activity of nsp14–nsp10. Increasing concentrations of nsp14–nsp10 were incubated with ss- or dsRNA with a blocked (3′-biotin) terminus (Oligo 2 and 16 see Supplementary Table 1A) and a fixed concentration of 300 nM nsp8 at (37°C, 45 min). Reactions were analysed by 20% denaturing PAGE. The SARS-CoV-2 nsp12–7–8 polymerase complex (at a 1:3:3 molar ratio) was incubated with 10 nM substrate (37°C, 5 min), prior to addition of 100 nM of nsp14–nsp10 at a 1:2 molar ratio of nsp14–nsp10 complex to nsp12–7–8 complex. Reactions were incubated at 37°C for 45 min and subsequently analysed by 20% denaturing PAGE. The size of products was determined as shown in Supplementary Figure 1E. Main products are labelled *, ** and *** corresponding to 12-mer, 10-mer and 7-mer respectively. Oligonucleotides used are given in Supplementary Table 1A and B.

To investigate which of the constituent polymerase subunits (i.e. nsp12, nsp7, and/or nsp8) mediates the stimulatory effect on nsp14–nsp10 activity, we tested each component alone and in combination on nsp14–nsp10 activity. Strikingly, nucleolytic stimulation of nsp14–nsp10 was clearly observed only in the presence of nsp8, regardless of whether other components (either one, or both, of nsp7 and nsp12) are present (Figure 4A).

To investigate whether the nsp14–nsp10 stimulation is mediated by, or is dependent on, the activity of the polymerase we also tested the effect of two mutant forms of nsp12: nsp12^D126A^, and nsp12^D760A/D761A^; the former being a NiRan domain active site mutant, and the latter a polymerase active site double mutant (22,23). Neither of these mutant forms of nsp12 affected the stimulation observed in the presence of nsp8 (Figure 4A). The stimulatory effect of nsp8 can be clearly observed in Figure 4B where the titration of nsp8 to the nuclease reaction results in a clear left shift in the pattern of nucleolytic digestion as the activity of nsp14–nsp10 becomes significantly more marked at lower concentrations (15 nM nsp14–nsp10) on both ss- and dsRNA.

To rule out the possibility that the stimulatory effect on nsp14–nsp10 catalysis caused by addition of nsp8 to nsp14–nsp10 was due to any intrinsic nsp8 nuclease activity, or a contaminant, we performed nuclease assays with isolated nsp12, nsp7, and nsp8. None of these showed any degradation of the RNA substrate, confirming that the nuclease activity observed (Figures 3 and 4) was due to nsp14–nsp10 activity (Supplementary Figure 3B).

Since we only observed the nsp14–nsp10 endonuclease activity (Figure 2D) at relatively high enzyme concentrations, we investigated whether nsp8 can stimulate this activity, by incubating increasing concentrations of nsp14–nsp10 with a fixed concentration of nsp8 with ss- and dsRNA substrates bearing a blocked (3′-biotin) terminus. Clear enhancement of nsp14–nsp10 dsRNA endonuclease activity was observed on addition of nsp8, even at the lowest nsp14–nsp10 concentration employed (15 nM), which is comparable to the substrate concentration (10 nM) (Figure 4C). Without nsp8, clear evidence of activity was not observed until the nsp14–nsp10 concentration was 250 nM. We conclude that nsp8 promotes the presence of all the identified nucleolytic activities of nsp14–nsp10 (Figure 4).

To understand whether this stimulatory effect of nsp8 on the activity of nsp14–nsp10 is mediated *via* protein-protein interactions, we performed RNA binding assays and protein pull-down assays. The results suggest relatively weak interaction between nsp8 and nsp14–nsp10 when examined by pull-down experiments and SPR (Supplementary Figure 3C and Supplementary Figure 3D). We executed a (reciprocal) pull-down, exploiting nsp14–nsp10′s intrinsic Ni-NTA affinity (plausibly mediated through the multiple zinc-finger motifs present in the complex) where nsp8 was indeed pulled down when nsp14–nsp10 was used as bait on Ni-NTA beads (Supplementary Figure 3C, lanes c–d), though contaminants in the nsp8 preparation were also seen to interact with Ni-NTA (Supplementary Figure 3C, lanes g–h), complicating interpretation of the results. Given the abolition of the nsp8 band (Supplementary Figure 3C, compare lanes g and h) when run alone upon 500 mM NaCl wash, but not in similar conditions with nsp14–nsp10, we infer that nsp8 binds to nsp14–nsp10, though this is a weak interaction. To further probe the interaction between nsp14–nsp10 and nsp8, we performed SPR (Supplementary Figure 3D). When nsp14–nsp10 was immobilised, we observed binding at increasing nsp8 concentrations in the absence of RNA. It should be noted that this interaction is relatively weak (Supplementary Figure 3D, calculated K_d_ = 8.3 × 10^−5^ M). Thus, the precise nature of the nsp8 and nsp14–nsp10 interaction, how it is mediated, and the biophysical effects should remain an area of further investigation (see Discussion section).

### Identification of nsp14–nsp10 inhibitors

The urgent need to identify therapeutics to treat COVID-19 led us to scope potential nsp14–nsp10 inhibitors, with a focus on compounds identified as potential COVID-19 treatments and known nuclease inhibitors. Previous studies suggest that inactivation of nsp14–nsp10 can lead to increased genomic instability and therefore a potentially lethal mutagenesis burden and concomitant reduction in viral propagation. While this alone might be insufficient to treat COVID-19 effectively, the possibility that nsp14–nsp10 inhibitors could be combined with inhibitors of other key factors (for example, the major proteases 3CL^pro^/M^pro^ or replicative RNA polymerase catalytic subunit nsp12) merits exploration.

Based on the efforts of ourselves and others to identify inhibitors of RNA and DNA nucleases, we performed *in silico* docking experiments focusing on chemotypes known to inhibit nucleases. We employed the AutoDock Vina platform (24) to dock in-house compounds on the entire exposed surface (blind docking) and the surface centred on the active site of a structure of SARS-CoV nsp14–nsp10 (PDB: 5NFY (10)). Use of a homology model of SARS-CoV-2 nsp14–nsp10 created using PDB:5NFY was considered inappropriate due to the low resolution of this structure. The results revealed a range of pharmacophores potentially interacting with the enzyme (Supplementary Figure 4A). Two *N*-hydroxyimides (A-1, and its positional isomer, A-2) were identified as having potential to interact with the nsp14–nsp10 active site (Supplementary Figure 4A–D).

**Figure 5.**
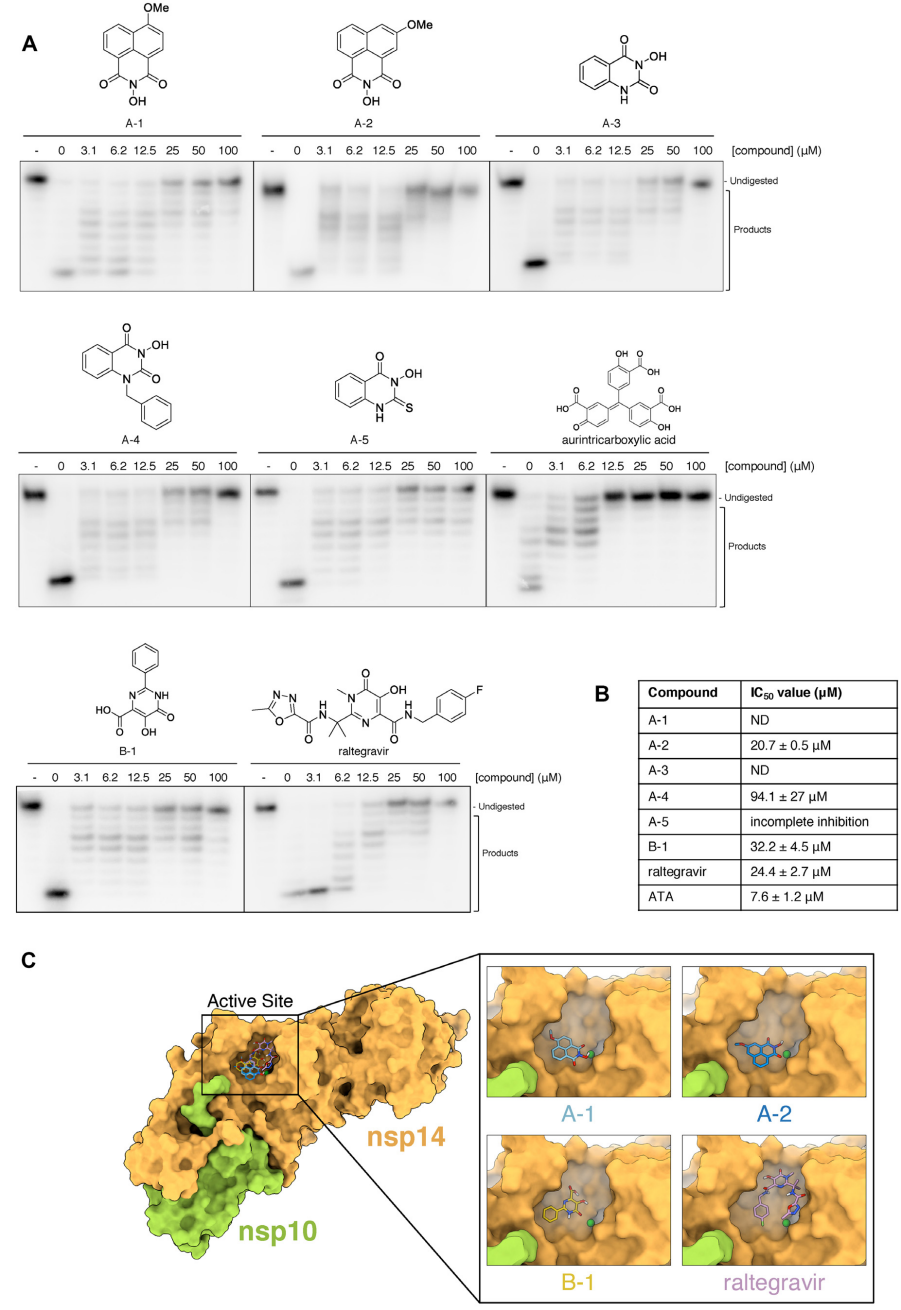
The exonuclease activity of nsp14–nsp10 is inhibited by *N*-hydroxyimide and hydroxypyrimidinone based compounds. (**A**) Increasing concentrations (as indicated, in μM) of compounds incubated with 100 nM nsp14–nsp10 (room temperature, 10 min), before initiating nuclease reaction by addition of ssRNA (37 °C, 45 min). Products were analysed by 20% denaturing PAGE. A decrease in the generation of nucleolytic reaction products and an increase in undigested substrate indicates inhibition of nuclease activity at increasing inhibitor concentrations. - indicates no enzyme. Compounds A-1–A-4 are based on a *N-*hydroxyimide scaffold, B-1 is a hydroxypyrimidinone. (**B**) IC_50_ values determined by quantification of gel digestion products (100 nM nsp14–nsp10); dose-response curves were determined by nonlinear regression. The mean ± s.e.m. were calculated from ≥3 biological repeats. (**C**) Docking of nsp14–nsp10 using Autodock. Nsp14–nsp10 was docked with compounds within grid boxes encompassing a surface focussed on the active site surface then the highest-affinity docking pose of A-1 and A-2 overlaid on the surface of SARS-CoV nsp14–nsp10; Mg^2+^ is in dark green and the highest-affinity docking poses of B1 and raltegravir overlaid on the surface of SARS-CoV nsp14–nsp10. Nsp14 is in yellow-orange, nsp10 is in light green, Mg^2+^ is in dark green. The docked poses of the compounds on the surface of the whole nsp14–nsp10 complex are shown on the left-hand side, with a detailed view at the active site on the right-hand side inset.

Efforts to establish a quantitative fluorogenic assay for nsp14–nsp10 activity, analogous to those we and others have devised for endo- and exonucleases previously (25), have as yet been unsuccessful due to the exquisite sensitivity of the nsp14–nsp10 complex to substrate modification by all fluorescence and quench groups tested. We therefore utilised a lower-throughput gel-based nuclease assay employing the 20-mer ssRNA described above to examine the potential inhibitory characteristics of compounds A-1 and A-2, available to us from existing chemical libraries. Aurintricarboxylic acid (ATA), which is a promiscuous ribonuclease inhibitor used during nucleic acid extraction protocols was used as a positive control (26), which as expected, inhibited nsp14–nsp10 with an IC_50_ of 7.6 ± 1.1 μM (100 nM nsp14–nsp10) (Figure 5; Supplementary Figure 5A). Figure 5A shows the inhibitory characteristics of A-1 and A-2 *N*-hydroxyimides in the gel-based assay, each of which exhibit qualitatively complete inhibition of substrate digestion. Quantification of the gel-based nuclease assay data did not yield an IC_50_ value for A-1, however, for A-2 this was determined to be 20 ± 0.5 μM (Figure 5B, Supplementary Figure 5A). Based on the docking data and potency of inhibition in the gel-based assay, we synthesised three structural variants of A-1 and A-2 (Supplementary Table 3). We decided to remove one of the rings, and use a bicyclic hydroxyimide scaffold, as has previously been used for inhibition of the nucleases FEN1 and XPF-ERCC1 (27,28), giving A-3 and A-4. We also attempted to introduce a thiocarbonyl group (A-5) to examine the potential effects on inhibition. However, none of these compounds exhibited lower IC_50_s than A-2 (Figures 5A and B; Supplementary Figure 5A).

The *N*-hydroxyimide pharmacophore is reported to inhibit structure-selective DNA nucleases, *via* chelation of an active site metal ion(s), in particular Mg^2+^ (27,28). Our *in silico* docking shows potential for A-1 and A-2 binding at the nsp14–nsp10 active site, with polar inhibitor atoms positioned close to the proposed catalytic Mg^2+^; wherein (for A-1) two of the hydroxyimide oxygen atoms are at distances of 2.4 and 2.6 Å from the Mg^2+^ (Figure 5C). Taking advantage of previous identification and detailed characterization of a series of FEN1 inhibitors, based around the *N*-hydroxyimide scaffold (29), we tested two compounds, AZ1353160 (denoted AZ-A1) and AZ13623940 (AZ-B1). Whilst some inhibition was observed, their potencies were also inferior to that of A-2 (Supplementary Figure 5B).

*N*-Hydroxypyrimidinones and hydroxypyrimidinones are structurally similar to the *N*-hydroxyimide pharmacophore and likely also inhibit nucleases through binding to the active site metal ion(s), typically Mg^2+^ (30,31). 5,6-Dihydroxyl-2-phenylpyrimidine-4-carboxylic acid (B-1) exhibited a comparable inhibition profile to A-2 with an IC_50_ value of 32.2 ± 4.5 μM (Figure 5A and B; Supplementary Figure 5A). We also tested raltegravir, an HIV integrase inhibitor, which contains a hydroxypyrimidinone ring (30,31). Similarly to B-1, raltegravir exhibited clear inhibition of the exonuclease activity of nsp14–nsp10 with an IC_50_ value of 24.4 ± 2.7 μM (Figure 5A & B; Supplementary Figure 5A). Our *in-silico* approach modelled B-1 docked proximal to the active site of nsp14–nsp10, with an oxygen atom of the hydroxypyrimidinone coordinating the catalytic Mg^2+^, at a distance of 2.7 Å (Figure 5C). The docked pose of raltegravir differed from that of B-1, however, it was again positioned close to the active site Mg^2+^, making additional contacts within the putative substrate binding pocket (Figure 5C).

**Figure 6.**
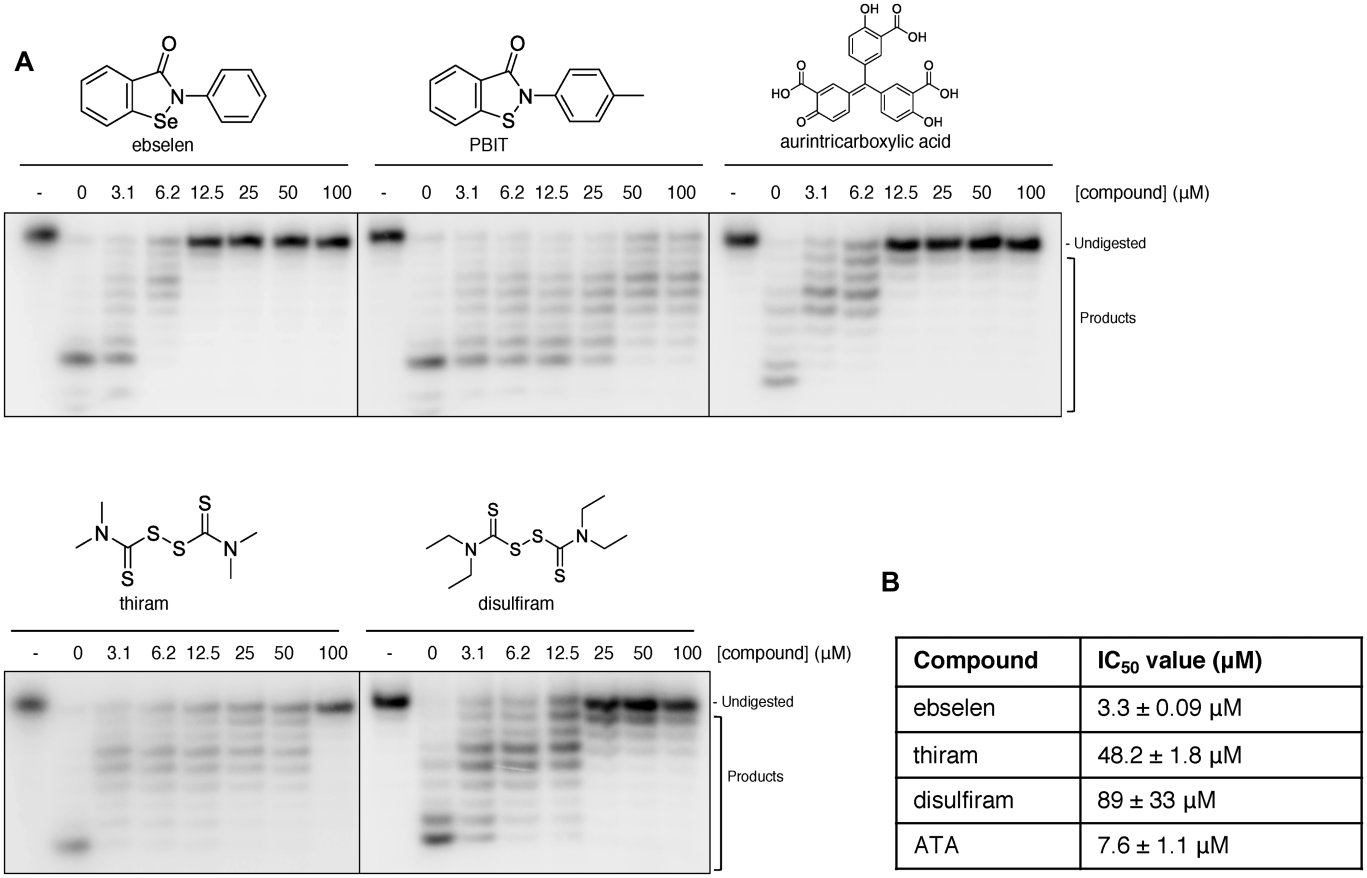
The exonuclease activity of nsp14–nsp10 is inhibited by the presence of drugs and drug-like compounds. (**A**) Increasing concentrations (as indicated, in μM) of drug and drug-like compounds were incubated with 100 nM nsp14–nsp10 (room temperature, 10 min), before initiation of nuclease reaction by the addition of ssRNA (37°C, 45 min). Products were analysed by 20% denaturing PAGE. A decrease in the generation of nucleolytic reaction products and a concomitant increase in undigested substrate indicates inhibition of nuclease activity. – indicates no enzyme. Gels are representative of at least three biological repeats. (**B**) IC_50_ values as calculated by quantification of gel digestion products (100 nM nsp14–nsp10) and dose-response curves were determined by nonlinear regression. The mean ± s.e.m. were calculated from ≥3 biological repeats. Precise IC_50_ values should be regarded as preliminary due to the nature of the assay.

These results provide potential pharmacophores that could be further developed and tested as COVID-19 treatments. However, the urgency of addressing COVID-19 requires that experimental or approved drugs can be repurposed for treatment. We therefore undertook at low-throughput (limited by the lack of a scalable fluorescent assay), a screen of 19 candidate drugs that might be predicted, on the basis of their mechanism of action, to inhibit the nuclease activity of nsp14–nsp10. These included nucleoside analogues, topoisomerase poisons, candidate DNA repair enzyme inhibitors, compounds reported to inhibit other COVID-19 targets, and antivirals believed to interfere with nucleic acid metabolism (Supplementary Table 2). Of these, ebselen (an organoselenium molecule with broad pharmacological properties (32) and a known potent inhibitor of the main CoV protease Mpro (33)) and disulfuram (a carbothioamide used to treat alcohol dependence) appeared most active in our screen. Detailed analysis allowed us to determine that the IC_50_ values for ebselen and disulfuram are 3.3 ± 0.09 and 89 ± 33 μM, respectively (Figure 6 and Supplementary Figure 5A). We also included thiram, a non-drug compound (used as a fungicide) which is also a carbothioamide closely related to disulfuram to determine whether this chemotype more generally acts to inhibit nsp14–nsp10. The IC_50_ for thiram was 48.2 ± 1.8 μM, suggesting that further surveys of carbothioamides are warranted in the search for nsp14–nsp10 inhibitors.

For the three of the most potent nsp14–nsp10 inhibitors identified (ebselen, A-2 and B-1) we also assessed whether inhibition was observed in the context of nsp8-mediated nuclease stimulation described above. Assays where nsp14–nsp10 is co-incubated with nsp8 revealed that inhibition was still observed, albeit at higher inhibitor concentrations consistent with the enhanced nuclease activity of the nsp14–nsp10 complex in the presence of nsp8 (Supplementary Figure 5C).

To investigate whether the compounds that exhibited inhibitory effects were not exerting these through aggregation or precipitation of nsp14–nsp10, we employed differential scanning fluorimetry (DSF) under identical reaction conditions to the inhibitor nuclease assays. None of the inhibitors showed any destabilizing effect on thermal stability of nsp14–nsp10, with the exception of ATA at higher concentrations (Supplementary Figure 5D and E).

Ebselen is reported to be generally tolerated in clinical studies investigating its effectiveness for treating a range of conditions from stroke and hearing loss to bipolar disorder. One mechanism of action proposed for ebselen relates to the potential capacity of the selenium to react with the thiolate ligands of zinc clusters in proteins and to release zinc (34–37). To test whether the selenium of ebselen is critical for the inhibition of nsp14–nsp10 nuclease activity, we investigated an analogue of it, PBIT, where a sulphur atom replaces the selenium of ebselen. Consistent with an important role for the selenium in nsp14–nsp10 inhibition, PBIT demonstrated dramatically reduced potency of inhibition (Figure 6A, IC_50_ not reached under assay conditions). We conclude that the best, potentially clinically useful nsp14–nsp10 inhibitor we have identified is the M^pro^ inhibitor ebselen, where the selenium moiety plays an important role mediating its inhibitory effects.

The compounds tested were screened for binding to SARS-CoV nsp14–nsp10 using AutoDock Vina; the relationship between the calculated affinities and potency (Supplementary Figure 4A–D and Supplementary Table 2) was not robust, in part reflecting the likely complex covalent inhibition mechanism by compounds such as ebselen. Interestingly, the majority of primary docked poses were in the nsp14 ExoN-MTase domain boundary, in a region otherwise occupied by substrates of the methyltransferase reaction, G5′ppp5′A and *S*-adenosyl methionine (PDB: 5C8S) (Supplementary Figure 4A–D) (8). While compounds docked at this site are unlikely to directly impact on competitive inhibition of nsp14–nsp10 activity, this result suggests that the MTase active site may also be a target.

We determined the capacity of three of the most potent nuclease inhibitors that docked in the proximity of the MTase in our simulations (ebselen, A-2 and B-1) to inhibit the guanine N7 methyl transferase (MTase) activity of nsp14–nsp10 using the homologous time-resolved fluorescence (HTRF) assay that monitors the activity of *S*-adenosyl methionine (SAM)-dependent methyltransferases (Supplementary Figure 5F). All three inhibitors, to variable degrees, demonstrated MTase inhibition activity, with B-1 being most potent compound. This observation raises the possibility that dual inhibition of both nuclease and MTase activities of nsp14–nsp10 could be achieved using compounds derived from the three chemotypes reported (Supplementary Figure 5G).

## DISCUSSION

Although the 3′-exonuclease activities of the SARS-CoV and SARS-CoV-2 are established, previous studies have primarily focused on structural aspects of the SARS-CoV-2 nsp14–nsp10 (38,39) and related SARS-CoV complexes (8,10). Our combined results comprise the first detailed biochemical analysis of the nsp14–nsp10 complex, that plays a key role in the genome duplication of the SARS-CoV-2 virus. The results confirm the presence of a 3′-exonuclease activity in the SARS-CoV-2 nsp14–nsp10 complex, which is curtailed when the 3′-hydroxyl terminus of the RNA substrate is derivatised (to a biotin group). Altering the nature of the substrate 3′-terminus, however, revealed a further feature of nsp14–nsp10 activity, namely its capacity to act as an endonuclease that catalyses incisions (closer to the 5′-end) of the substrate. We observed a robust and almost equivalent nuclease activity of nsp14–nsp10 on both ssRNA and dsRNA. We also observed efficient excision of terminally mismatched nucleotides by nsp14–nsp10 from SARS-CoV-2, but where mismatches longer than 2- or 3- ribonucleotides are less efficiently excised, as has been reported for the analogous SARS-CoV complex (3).

The nsp14–nsp10 exonuclease activity is capable of degrading (approximately) the first eight ribonucleotides from the 3′-end of all substrates tested (either ssRNA or dsRNA). When the 3′-terminus is blocked with a biotin group, nsp14–nsp10 acts further downstream (5′-to) to these sites to endonucleolytically release a fragment of predominantly 10-ribonucleotides. We therefore propose that the role of nsp14–nsp10 has potential to extend beyond that of a proof-reader, by analogy with the 3′-exonuclease present in replicative DNA polymerase complexes (11). nsp14–nsp10 may represent a simple replication-repair system that removes ribonucleotides from the 3′-end of elongating nascent replicating RNA strand when extension is blocked. It is likely that nsp14–nsp10 is constitutively active during viral replication. It is possible that only when replication stalls (due to mismatch incorporation or the presence of altered ribonucleotides that cannot be further extended by the polymerase), that nsp14–nsp10 acts to degrade and/or cleave the elongating nascent strand, so removing the aberration and allowing for re-engagement of the RNA polymerase and resumption of synthesis.

It has recently been reported that nsp14 and nsp10 are required for recombination between CoV genomes, and this might represent a strategy for rescuing viral genomes where replication is perturbed (40). Indeed, nsp14 (ExoN)-deficient SARS-CoV-2 virus is more sensitive to several replication terminating nucleoside analogues including remdesivir and β-D-N^4^-hydroxycytidine (NHC, the active species produced from the prodrug molnupiravir) and is susceptible to lethal mutagenesis by several of these agents (5,41,42).

Together, our observations and those reported elsewhere support the assertion that nsp14–nsp10 acts to remove a broad spectrum of chain terminating damage during viral replication; its versatile catalytic nature comprising both exo- and endo-nucleolytic functions means it is well-adapted to this role. We note that an important feature of the nsp14–nsp10 activity is its insensitivity to common RNA modifications that are associated with viral evasion (6-methyladenine) or induction (inosine) of innate immune pathways; this may be an evolved property contributing to the robust virulence of the SARS-CoV-2.

Replication and repair in a viral genome are closely linked; our work showing that the addition of the polymerase complex promotes nsp14–nsp10 nuclease activity raises the question of the relationship between the two activities. Importantly, the presence of the polymerase complex nsp12–7–8 enhances the activity of nsp14–nsp10 on a variety of different substrates; this was particularly so for uracil-containing substrates, which may be of relevance when considering the physiological life-cycle of the virus. Notably, nsp14–nsp10 alone poorly digests homopolymeric RNA substrates, including poly(U), whereas in the presence of nsp12–nsp7–nsp8 efficient processing of poly(U) in both single-stranded form and as a duplex annealed to poly(A) is observed. Strikingly, the processing of other homopolymers examined (poly(G) and poly(C)) was not enhanced by the addition of nsp12–7–8, implying that it may contribute to nsp14–nsp10 substrate selectivity *in vivo*. The 3′-end of the plus strand of CoV genomes is polyadenylated by between 100 and 130 A-residues (43). Poly(U–A) duplexes could therefore accumulate during the replication of CoV by RdRp, by virtue of the generation of a complementary poly(U) tract at the 5′-end of the nascent minus strand (1). This dsRNA has been proposed to constitute a pathogenic-associated molecular pattern (PAMP) and to initiate an innate immune response following recognition by MDA5 (43). One function of nsp14–nsp10 in the context of ongoing replication might be to limit the accumulation poly(U-A) to restrain innate immune signalling. Notably, a similar role has been proposed for CoV nsp15, which is an endoribonuclease (endoU) (43) and the possibility of interplay and co-operation of nsp14–nsp10 with this factor is worthy of further examination.

This enhancement of both exo- and endo-nucleoytic nsp14–nsp10 activity was specifically attributed to the nsp8 subunit of the nsp12–7–8 polymerase complex. An interaction between nsp12 and nsp14 has previously been reported for SARS-CoV using GST pull-down assays (10); however, we were unable to detect this for the analogous SARS-CoV-2 proteins. We note that nsp12 alone is incapable of stimulating nsp14 nuclease activity. While the stimulation of nsp14–nsp10 could be observed by addition of nsp8 alone to nuclease reactions, we were unable to observe more than a weak interaction between nsp14 and nsp8. Several lines of evidence (yeast-two hybrid analysis and immunoprecipitation) suggest that nsp8 interacts promiscuously with multiple SARS-CoV nsps, including nsp14 and nsp10 (44), while nsp14–nsp10 might have additional interactions that mediate its activity (45).

Structural studies which have revealed that nsp8 in the replisome contains positively charged ‘sliding poles’ which extend from the main body of the nsp12-nsp7-nsp8 complex (23,46,47). These reach up to 28 ribonucleotides from the nsp12 active site, in the SARS-CoV-2 RdRp complex (46). The sliding poles are flexible in the absence of RNA, become ordered upon engagement with RNA, and are required for the formation of a highly processive replisome required to replicate the long SARS-CoV genomes (46). The sliding poles might act as a platform to mediate the range of nsp interactions reported, possibly through its presentation of an accessible positively charged surface. Extensive attempts to observe the co-operative association of nsp8 and nsp14 (and nsp14–nsp10) in the presence of a variety of RNA substrates using electrophoretic-mobility shift assays (EMSA) did not provide evidence for the formation of a stable super-complex (data not shown). It is nonetheless likely that relatively weak (transient) interactions between nsp8, potentially mediated by positively charged nsp8 poles, are sufficient to allosterically stimulate nsp14 activity or sufficiently increase the half-life of nsp14–RNA association. Another possibility is that nsp8–RNA engagement and a subsequent conformational change of the RNA bound nsp8 poles modulates substrate conformation in a manner that renders the RNA more amenable to digestion. Other workers have recently reported that nsp8 and nsp14–nsp10 co-elute during SEC, but they were unable to demonstrate a robust interaction between the nsp8 and the ExoN complex in cryo-EM studies, and draw similar conclusions to ourselves about the structural plasticity of nsp8 which is modulated in the context of the RNA-dependent RNA polymerase complex (RdRp) and while engaged with its substrate (39). Interestingly, the authors of a recent structural (cryo-EM) study which elucidated the structure of the nsp14–nsp10-containing replisome proposed that their data inferred that proofreading might occur in *trans*, with the damaged nascent strand associated with the stalled replication complex being processed by nsp14–nsp10 present at another replisome (48). This scenario would add further complexity to understanding of the mechanism of nsp14–nsp10 mediated repair and fork restart and the role of nsp8; it is of interest to test this possibility in a more completely-reconstituted replication-repair system.

Due to the urgency of identify new agents to treat COVID-19, we performed an *in-silico* docking screen to identify chemotypes capable of interacting with the metal-containing active site of nsp14 and a focused screen of drugs (approved and in development) to identify compounds that might inhibit nsp14–nsp10. Our docking simulations, employing structural data for the highly homologous SARS-CoV nsp14–nsp10 complex, revealed that *N-*hydroxyimide based compounds could potentially interact with the active site magnesium ion. Testing of this possibility revealed one compound (A-2) with an IC_50_ of 20.7 ± 0.5 μM (with 100 nM nsp14–nsp10). In the limited time available, we generated several chemical variants around this pharmacophore, but none to date out-performed A-2, as was the case for two related compounds previously developed to inhibit the FEN1 DNA nuclease (29). We expanded our search to explore the putative inhibitory potential of the related hydroxypyrimidone scaffold. Compound B-1 exhibited comparable levels of inhibition to the *N-*hydroxyimide based compounds, as did the HIV integrase inhibitor, raltegravir, with IC_50_ values of 32.3 ± 4.5 and 24.4 ± 2.7 μM, respectively.

We modelled possible binding models of A-1, A3–5, B-1 and raltegravir to nsp14–nsp10. In accord with previous literature, the results show the *N-*hydroxyimide and hydroxypyrimidone scaffolds have the potential to chelate the active site magnesium ion of the SARS-CoV nsp14–nsp10 (27,29), providing insight into the possible mode of inhibition. Interestingly, modelling with raltegravir suggests this larger compound could be accommodated in the active site pocket, potentially making additional contacts, which may be useful in the generation of selective inhibitors of nsp14–nsp10.

Our focused screens of drug and drug candidates revealed that the selenium-containing drug ebselen is an effective inhibitor of nsp14–nsp10. Ebselen is a potent inhibitor of the major SARS-CoV protease (M^pro^), and manifests antiviral activity in cell-based assays (33). The potencies of ebselen for nsp14–nsp10 and M^pro^ inhibition are similar *in vitro*; the IC_50_ for M^pro^ was 4.67 μM, and for nsp14–nsp10 we determined an IC_50_ of 3.3 μM. The mechanism(s) of action of ebselen in nsp14–nsp10 inhibition remains unknown, but given the known role for the selenium in ebselen in the co-ordination or ejection of zinc atoms in these proteins (35–37), we propose this is one possible mode of action. Indeed, zinc fingers are highly represented in the proteome of SARS-CoV-2 (33), where the M^pro^ possesses zinc binding motifs and the SARS-CoV-2 nsp10 possesses at least one zinc finger which is likely to be important for mediating interactions with the RNA viral genome (8,10). Interference with these zinc atoms from these binding motifs might induce conformational changes that are not compatible with robust activity. However, it should also be noted that ebselen reacts multiple times with M^pro^, like *via* reaction with cysteines, in addition to those at the active site (49). Whatever the precise mechanisms of action of ebselen, given that it is capable of targeting two key enzymes required for the propagation of SARS-CoV-2, it is reasonable that further studies assessing the utility of this agent in combatting COVID-19, as a stand-alone agent or in combination, are warranted. It should also be noted that ebselen exhibited antiviral activity in cell-based assays (33), whereas the less potent nsp14–nsp10 inhibitor raltegravir did not (50). The activity of ebselen might plausibly be attributed to attenuating the activity of several key SARS-CoV-2 proteins including M^pro^ and nsp14–nsp10. More generally, given that many important antimicrobial medicines act by binding to multiple targets (e.g. β-lactams such as penicillins and cephalosporins), we suggest that polypharmacology be pursued as a matter of priority for COVID-19 treatment.

## DATA AVAILABILITY

The constructs and full data presented in this study are available upon request.

## Supplementary Material

gkab1303_Supplemental_Files

## Notes

Present address: Opher Gileadi, Exscientia, The Schrödinger Building Oxford Science Park, Oxford OX4 4GE, UK.

Present address: Yuliana Yosaatmadja, School of Biological Sciences, Building 110, 3A Symonds Street, Auckland 1010, New Zealand.
