# Supplementary material for: Characterization of the SARS-CoV-2 ExoN (nsp14ExoN–nsp10) complex: implications for its role in viral genome stability and inhibitor identification
Source: Nucleic Acids Res. 2022 Jan 17;50(3):1484–500. doi: 10.1093/nar/gkab1303 (PMC8860572; doi:10.1093/nar/gkab1303)
Supplement: gkab1303_Supplemental_Files [file gkab1303_supplemental_files.zip › Baddock-Supplemental M&M .pdf]

SUPPLEMENTAL MATERIALS AND METHODS

Cloning and site directed mutagenesis of wildtype and nuclease dead (NSP14 $^{D113A/E115A}$) nsp14-nsp10 complex.

Wildtype (WT) NSP14 and NSP10 encoding DNA were codon-optimised for *Escherichia coli (E. coli)* expression and were synthesised by Twist Bioscience (California, USA). Both the NSP14 and NSP10 genes were subcloned into the pNIC28-BSA4 vector[1] to enable bicistronic expression and appropriately stoichiometric production of nsp14 and nsp10 proteins.

Site-directed-mutagenesis (SDM) was carried out using an 'inverse' PCR experiment, whereby an entire plasmid is amplified using complementary mutagenic primers with minimal cloning steps[2]. The Herculase II Fusion DNA Polymerase (Agilent) was utilised and SDM PCR was performed to amplify the whole plasmid, according to the manufacturer's instructions. The PCR product was then added to a standard KLD enzyme mix (NEB) reaction and was incubated at room temperature for 1 hour, prior to transformation into chemically competent *E. coli* cells.

Immunoprecipitation experiments between nsp12, nsp7, nsp 8, and nsp14

Relevant nsp combinations were mixed together in buffer P (25 mM HEPES (pH 7.5), 50 mM NaCl, 5% glycerol, 1 mM DTT, 0.05% Triton X-100, 10 mM $MgCl_2$), and allowed to form a complex for 5 min at 37 °C prior to incubating with Ni-NTA Agarose (Qiagen) beads for 2 h at 4 °C with tumbling. The beads were then washed thrice with buffer P + 150 mM NaCl and half of this sample with buffer P + 500 mM NaCl. Bound proteins were eluted from the beads by boiling with SDS, then analysed by SDS-PAGE.

Surface plasmon resonance (SPR) with nsp14, nsp14-10, and nsp8

For binding studies involving nsp8 and nsp14-nsp10, a Biacore T200 instrument (RCaH, Harwell, UK) equipped with a CM5 sensor chip (GE Healthcare) was used. All samples and buffers were filtered prior to use.

Nsp14-nsp10 proteins were immobilized on a CM5 chip (GE Healthcare) using an amide coupling kit: standard 1-ethyl-3-(3-dimethylaminopropyl)carbodiimide /N-hydroxysuccinimide mediated coupling was performed. For nsp14-nsp10 immobilization, a 50 µg mL^{-1} solution in 10 mM acetate acid (pH 5.5) was allowed to react with the activated ester surface for 60 seconds, allowing an immobilisation ~4000 response units (RU) on each flow cell. The surface was washed using HEPES running buffer (25 mM HEPES pH 7.5, 50 mM NaCl, 5% glycerol, 1 mM TCEP, 0.05% Triton and 10 mM $MgCl_2$). After immobilization, the remaining N-hydroxysuccinimide esters were blocked by the addition of 1 M ethanolamine to the sensor, in both sample and reference cells. Nsp8 protein was injected at increasing concentrations (0, 0.625, 1.25, 2.5, 5, 10 and 20 µM) for 120 seconds at 30 µL/min and dissociation monitored for 300 seconds.

Data were referenced, solvent corrected, and processed using the T200 evaluation software.

Compound synthesis

General Methods

All reagents were purchased from Sigma-Aldrich, Acros Organics, Fluka, Fluorochem, Abcr, and Fisher Scientific and used without further purification. Microwave reactions were performed using an Initiator 2 microwave reactor (Biotage). Flash column chromatography was performed on a Biotage Isolera automated flash column chromatography platform using Biotage Sfär or Biotage SNAP Ultra columns.

IR spectra were recorded on a Bruker Tensor 27 FT-IR spectrometer as a solid or thin film. Selected characteristic peaks are reported in cm^{-1}. NMR spectra were recorded using Bruker Avance spectrometers in the deuterated solvent stated. Assignments given correspond with the numbering system as drawn; where assignments are not provided, it was impossible to assign the resonances due to overlapping signals. Low-resolution mass spectra were recorded on an Agilent Technologies 1260 Infinity LC-MS system fitted with a 6120 Quadrupole mass spectrometer. High-resolution

mass spectra (HRMS) were recorded in HPLC grade methanol using electrospray ionisation (ESI+/-) on a Bruker APEX III FT-ICR mass spectrometer.

2-Benzyloxy-6-bromo-benzo[de]isoquinoline-1,3-dione (1)

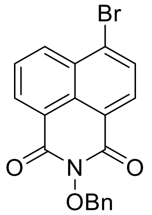

To a solution of 6-bromo-benzo[de]isochromene-1,3-dione (300 mg, 1.08 mmol, 1 equiv.) in anhydrous pyridine (10 ml), *O*-benzylhydroxylamine hydrochloride (346 mg, 2.16 mmol, 2 equiv.) was added. The reaction mixture was stirred under reflux for 2 h under an inert atmosphere. It was then cooled to RT, concentrated *in vacuo,* and EtOH (20 mL) was added to a solid residue. The precipitate was collected by filtration, washed with cold EtOH, and dried to give 2-benzyloxy-6-bromo-benzo[de]isoquinoline-1,3-dione (395 mg, 1.03 mmol, 96%) as orange solid. IR v_{max}/cm^{-1} (solid): 1719 and 1677 (C=O); ^{1}H NMR (400 MHz, $CDCl_3$) δ 8.63 (dd, *J* = 7.3, 1.2 Hz, 1H), 8.55 (dd, *J* = 8.5, 1.2 Hz, 1H), 8.38 (d, *J* = 7.9 Hz, 1H) 8.00 (d, *J* = 7.9 Hz, 1H), 7.81 (dd, *J* = 8.5, 7.3 Hz, 1H.), 7.66 – 7.57 (m, 2H), 7.39 – 7.28 (m, 3H), 5.20 (s, 2H); ^{13}C NMR (101 MHz, $CDCl_3$) δ 160.53, 133.99, 133.94, 132.51, 131.60, 131.35, 131.12, 130.90, 130.02, 129.21, 128.54, 128.32, 128.30, 123.31, 122.42, 78.70; LRMS: *m/z*(%)= 382.2 (100), 384.2 (98) $[M + H]^{+}$; HRMS: calcd. $C_{19}H_{13}O_3N^{79}Br$ $[M + H]^{+}$: 382.0073; found: 382.0070.

2-Benzyloxy-6-methoxy-benzo[de]isoquinoline-1,3-dione (2)

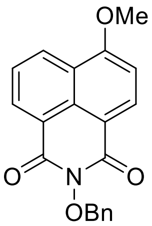

To a solution of 1 (360 mg, 0.94 mmol, 1 equiv.) and $CuSO_4$ (10 mg, 0.06 mmol, 0.06 equiv.) in anhydrous MeOH (8 mL), NaOMe 25 wt. % in MeOH (0.27 mL, 1.43mmol,

1.5 equiv.) was added. The reaction mixture was stirred under reflux for 12 h under an inert atmosphere. It was then cooled to RT. The precipitate was collected by filtration, washed with H_2O, and dried to give 2-benzyloxy-6-methoxy-benzo[de]isoquinoline-1,3-dione (286 mg, 0.86 mmol, 90%) as a yellow solid. IR ν_{max}/cm^{-1}(solid): 1770 and 1743 (C=O); ^{1}H NMR (400 MHz, $CDCl_3$) δ 8.64 (dd, *J* = 7.3, 1.3 Hz, 1H), δ 8.60 (dd, *J* = 8.4, 1.2 Hz, 1H), 8.59 (d, *J* = 8.3 Hz, 1H), 7.72 (dd, *J* = 8.4, 7.3 Hz, 1H), 7.69 (dd, *J* = 7.8, 1.7 Hz, 2H), 7.46 – 7.33 (m, 3H), 7.07 (d, *J* = 8.3 Hz, 1H), 5.26 (s, 2H), 4.14 (s, 3H); ^{13}C NMR (101 MHz, $CDCl_3$) δ 161.49, 161.47, 161.00, 134.40, 134.09, 132.09, 130.12, 129.42, 129.16, 128.82, 128.61, 126.24, 123.91, 122.70, 115.19, 105.58, 78.64, 56.48.; LRMS: *m/z*(%)= 334.2 (100) $[M + H]^+$; HRMS: calcd. $C_{20}H_{16}O_4N$ $[M + H]^+$: 334.1074; found: 334.1075.

2-Hydroxy-6-methoxy-benzo[de]isoquinoline-1,3-dione (3)

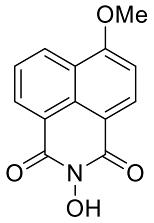

To a solution of 2 (275 mg, 0.83 mmol, 1 equiv.) in DMA (10 mL), palladium on carbon (35 mg, 10 wt. %) was added. The reaction mixture was stirred for 4 h at RT under a hydrogen atmosphere. The reaction was filtered through celite, washed with DMA, and concentrated *in vacuo*. The solid residue was then washed with MeOH and dried to give 2-hydroxy-6-methoxy-benzo[de]isoquinoline-1,3-dione (194 mg, 0.80 mmol, 96%) as a yellow solid. Melting Point (from EtOH): 259-261°C; IR ν_{max}/cm^{-1}(solid): 1703 and 1650 (C=O); ^{1}H NMR (400 MHz, DMSO-d_6) δ 10.63 (brs, 1H), 8.51 (app. ddd, *J* = 8.5, 7.8, 1.2 Hz, 2H), 8.46 (d, *J* = 8.3 Hz, 1H), 7.81 (dd, *J* = 8.4, 7.3 Hz, 1H), 7.32 (d, *J* = 8.4 Hz, 1H), 4.13 (s, 3H); ^{13}C NMR (101 MHz, DMSO-d_6) δ 160.91, 160.55, 160.48, 133.42, 131.13, 128.47, 127.44, 126.51, 122.90, 122.20, 114.40, 106.43, 56.70; LRMS: *m/z*(%)= 244.1 (100) $[M + H]^+$; *HRMS:* calcd. $C_{13}H_9O_4N^{23}Na$ $[M + Na]^+$: 266.04238; found: 266.04261.

5-Methoxy-benzo[de]isochromene-1,3-dione (4)

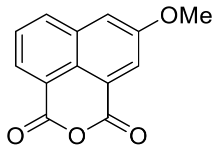

To a solution 5-hydroxy-benzo[de]isochromene-1,3-dione (100 mg. 0.46 mmol, 1 equiv.) in anhydrous acetone (5 mL), SO_4Me_2 (0.8 mL, 0.93 mmol, 2 equiv.) and K_2CO_3 (200 mg, 1.45 mmol, 3 equiv.) were added. The reaction mixture was stirred under reflux for 3 h under inert atmosphere and concentrated *in vacuo*. Then 1 M $HCl_{(aq)}$ (20 mL) was added, precipitate was filtered, washed with H_2O and dried to give 5-methoxy-benzo[de]isochromene-1,3-dione (78 mg, 0.34 mmol, 73%) as a cream solid. IR v_{max}/cm^{-1}(solid): 1774 and 1739 (C=O) ^{1}H NMR (400 MHz, DMSO-d_6) δ 8.42 (dd, J = 8.3, 1.1 Hz, 1H), 8.35 (dd, J = 7.3, 1.1 Hz, 1H), 8.06 (d, J = 2.5 Hz, 1H), 8.02 (d, J = 2.6 Hz, 1H), 7.86 (dd, J = 8.3, 7.3 Hz, 1H), 4.01 (s, 3H); ^{13}C NMR (101 MHz, DMSO-d_6) δ 161.1, 160.8, 158.3, 134.5, 133.7, 130.2, 128.5, 125.6, 123.7, 121.0, 119.4, 114.8, 56.6; LRMS: *m/z*(%)= 229.1 (100) $[M + H]^+$; HRMS: calcd. $C_{13}H_9O_4$ $[M + H]^+$: 229.0495; found: 229.0498;

2-Hydroxy-5-methoxy-benzo[de]isoquinoline-1,3-dione (5)

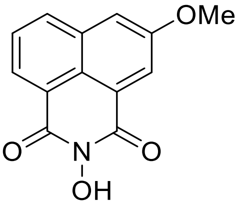

To a solution of 4 (78 mg, 0.34 mmol, 1 equiv.) in anhydrous pyridine (3 mL), $NH_2OH\cdot HCl$ (45 mg, 0.66 mmol, 2 equiv.) was added. The reaction mixture was stirred under reflux for 1 h under an inert atmosphere. The reaction mixture was then diluted with cold H_2O (75 mL). The precipitate was collected by filtration, washed with H_2O, and dried to give 2-hydroxy-5-methoxy-benzo[de]isoquinoline-1,3-dione (81 mg, 0.33, 97%) as a yellow solid. Melting Point (from EtOH): 233-236°C; IR v_{max}/cm^{-1}(solid): 1682 and 1628 (C=O); ^{1}H NMR (400 MHz, DMSO-d_6) δ 10.68 (brs, 1H, O(17)H) 8.31 (d, J = 7.5 Hz, 2H), 8.01 (d, J = 2.4 Hz, 1H), 7.89 (d, J = 2.6 Hz, 1H,), 7.80 (app. t, J = 7.7 Hz, 1H), 3.97 (s, 3H); ^{13}C NMR (101 MHz, DMSO-d_6) δ 161.36, 160.91,

158.20, 133.75, 133.67, 128.66, 128.17, 124.29, 122.61, 122.20, 122.11, 113.86, 56.45; LRMS: *m/z*(%)= 244.1 (100) $[M + H]^+$; HRMS: calcd. $C_{13}H_{10}O_4N$ $[M + H]^+$: 244.0604; found: 244.0606

2-Amino-N-(benzyloxy)benzamide (6)

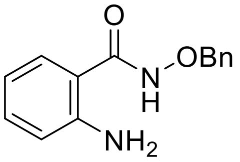

To a solution of Isatoic anhydride (6 g, 36.8 mmol, 1 equiv.) and *O*-benzylhydroxylamine hydrochloride (8.6 g, 54.0 mmol, 1.5 equiv.) in anhydrous THF (50 mL), TEA (7.5 mL, 54.0 mmol, 1.5 equiv.) was added. The reaction mixture was stirred under reflux for 3 h under an inert atmosphere and then concentrated *in vacuo*. The product was purified using flash column chromatography (10-30% EtOAc in cyclohexane) to give 2-amino-*N*-(benzyloxy)benzamide (7.8g, 32.2 mmol, 88%) as a white solid. IR v_{max}/cm^{-1}(solid): 1626 (C=O); ^1H NMR (400 MHz, DMSO-d_6) δ 11.65 (s, 1H), 7.96 (dd, *J* = 7.9, 1.5 Hz, 1H), 7.68 (ddd, *J* = 8.5, 7.2, 1.6 Hz, 1H), 7.60 – 7.53 (m, 2H), 7.48 – 7.34 (m, 3H), 7.30 – 7.19 (m, 2H) 5.10 (s, 2H); ^{13}C NMR (101 MHz, DMSO-d_6) δ 159.50, 148.53, 139.18, 135.56, 134.95, 129.93, 129.32, 128.84, 127.72, 123.17, 115.93, 114.91. 77.95; LRMS: *m/z*(%)= 243.1 (100) $[M + H]^+$; HRMS: calcd. $C_{14}H_{15}O_2N_2$ $[M + H]^+$: 243.1128; found: 243.1127;

3-Benzyloxyquinazoline-2,4-dione (7)

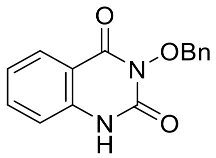

To a solution of 6 (1 g, 4,1 mmol, 1 equiv.) and triphosgene (1.35 g, 4.5 mmol 1,1 equiv.) in anhydrous THF (40 mL) at 0 °C, TEA (1.26 mL, 9.0 mmol, 2.2 equiv.) was added dropwise. The reaction mixture was stirred for 2 h at RT under an inert atmosphere and then concentrated *in vacuo*. The product was purified using flash column chromatography (10-60% EtOAc in cyclohexane) to give

3-benzyloxyquinazoline-2,4-dione (726 mg, 2.71 mmol, 66%) as a white solid. IR ν_{max}/cm^{-1}(solid): 1733 and 1664 (C=O); ^{1}H NMR (400 MHz, DMSO-d_6) δ 11.44 (s, 1H), 7.59 – 7.35 (m, 5H), 7.29 (dd, *J* = 8.0, 1.6 Hz, 1H), 7.15 (ddd, *J* = 8.6, 7.0, 1.6 Hz, 1H), 6.71 (d, *J* = 8.2 Hz, 1H), 6.49 (t, *J* = 7.5 Hz, 1H), 4.90 (s, 2H); ^{13}C NMR (101 MHz, DMSO-d_6) δ 167.68, 150.01, 136.52, 132.57, 129.33, 128.76, 128.71, 128.21, 116.79, 115.11, 112.81, 77.41; LRMS: *m/z*(%)= 269.1 (100) $[M + H]^+$; HRMS: calcd. $C_{15}H_{11}O_2N_3$ $[M - H]^-$: 267.0776; found: 267.0775;

3-Hydroxyquinazoline-2,4-dione (8)

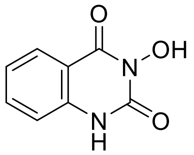

To a degassed solution of 7 (50 mg, 0.19 mmol, 1 equiv.) in DMF (2 mL), palladium on carbon (10 mg, 10 wt. %) was added. The reaction mixture was stirred under an H_2 atmosphere for 2 h. It was then filtered through celite and concentrated *in vacuo*. 3-Hydroxyquinazoline-2,4-dione (28 mg, 0.16 mmol, 87%) was gained as a white solid. IR ν_{max}/cm^{-1}(solid): 1714 and 1654 (C=O); ^{1}H NMR (400 MHz, DMSO-d_6) δ 11.05 (brs, 2H), 7.94 (d, *J* = 8.1 Hz, 1H), 7.66 (t, *J* = 7.7 Hz, 1H), 7.35 – 7.13 (m, 2H); ^{13}C NMR (101 MHz, DMSO-d_6) δ 159.87, 149.23, 138.84, 135.14, 127.53, 122.98, 115.75, 114.61; LRMS: *m/z*(%)= 179.1 (100) $[M + H]^+$; HRMS: calcd. $C_8H_5O_3N_2$ $[M - H]^-$: 177.0306; found: 177.0298;

1-Benzyl-3-benzyloxyquinazoline-2,4-dione (9)

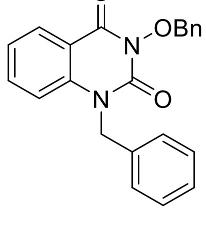

To a solution of 7 (200 mg, 0.75 mmol, 1 equiv.) and Cs_2CO_3 (485 mg, 1.5 mmol, 2 equiv.) in anhydrous DMF (3 mL), benzyl bromide (106 µL, 0.89 mmol, 1.2 equiv.) was

added. The reaction mixture was stirred at 80 °C for 2 h and quenched with cold H_2O (75 mL). The precipitate was collected by filtration, washed with H_2O, and dried under a vacuum. 1-benzyl-3-benzyloxyquinazoline-2,4-dione (256 mg, 0.72 mmol, 95%) was gained as a white solid. IR v_{max}/cm^{-1}(solid): 1720 and 1684 (C=O); ^1H NMR (400 MHz, $CDCl_3$) δ 8.26 (dd, *J* = 7.9, 1.7 Hz, 1H), 7.64 (dd, *J* = 6.5, 2.9 Hz, 2H), 7.55 (ddd, *J* = 8.8, 7.3, 1.7 Hz, 1H), 7.45 – 7.16 (m, 10H), 7.11 (d, *J* = 8.5 Hz, 1H), 5.37 (s, 2H), 5.29 (s, 2H); ^{13}C NMR (101 MHz, $CDCl_3$) δ 158.70, 149.68, 139.20, 135.24, 135.22,130.24, 129.18, 129.03, 128.99, 128.49, 127.82, 126.50, 123.42, 114.72, 78.43, 47.47; LRMS: *m/z*(%)= 359.2 (100) $[M + H]^+$, HRMS: calcd. $C_{22}H_{19}O_3N_2$ $[M + H]^+$: 359.1390; found: 359.1392;

1-Benzyl-3-hydroxyquinazoline-2,4-dione (10)

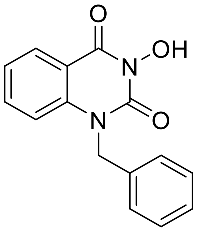

A mixture of 9 (240 mg, 0.67 mmol, 1 equiv.) in conc. HBr (48% in H_2O, 2 mL) and AcOH (2 mL) was stirred under reflux for 1 h. The reaction mixture was then concentrated *in vacuo*, dissolved in DMF (0.5 mL) and poured into cold H_2O (50 mL). The precipitate was then collected by filtration, washed with a minimal amount of cold DCM, and dried under vacuum. 1-Benzyl-3-hydroxyquinazoline-2,4-dione (51 mg, 0.19 mmol, 28%). IR v_{max}/cm^{-1}(solid): 1712 and 1666 (C=O); ^1H NMR (400 MHz, DMSO-d_6) δ 10.86 (s, 1H), 8.09 (dd, *J* = 7.8, 1.6 Hz, 1H), 7.67 (ddd, *J* = 8.7, 7.2, 1.7 Hz, 1H), 7.39 – 7.22 (m, 7H), 5.40 (s, 2H); ^{13}C NMR (101 MHz, DMSO-d_6) δ 158.96, 150.27, 139.13, 136.60, 135.44, 129.19, 128.23, 127.79, 126.91, 123.55, 115.70, 115.61, 46.87; LRMS: *m/z*(%)= 269.2 (100) $[M + H]^+$; HRMS: calcd. $C_{15}H_{13}O_3N_2$ $[M + H]^+$: 269.0921; found: 269.0922;

3-Hydroxy-2-thioxo-2,3-dihydroquinazolin-4-one (11)

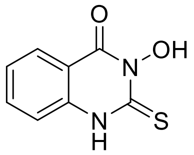

To a solution of methyl anthranilate (150 µL, 1.16 mmol, 1 equiv.) in H_2O (3 mL) and DCM (10 mL), thiophosgene (100 µL, 1.30 mmol, 1.1 equiv.) was added dropwise. The reaction mixture was stirred at RT for 16 h. It was then diluted with H_2O (15 mL) and extracted with DCM (3 × 15 mL). The combined organic layer was washed with brine (20 mL), dried over Na_2SO_4, and concentrated *in vacuo*. Intermediate methyl 2-isothiocyanatobenzoate was then added to a solution of NH_2OH·HCl (82 mg, 1.18 mmol, 1 equiv.) and sodium hydroxide (47 mg, 1.20 mmol, 1 equiv.) in a mixture of chloroform/H_2O (1:1, 10 mL). The reaction mixture was stirred for 2h at RT. The precipitate was collected by filtration, washed with DCM, and dried under vacuum. 3-Hydroxy-2-thioxo-2,3-dihydroquinazolin-4-one (179 mg, 0.92 mmol, 80%) was gained as a white solid. IR v_{max}/cm^{-1}(solid): 1701 (C=O) and 1619 (C=S); ^1H NMR (400 MHz, DMSO-d_6) δ 12.97 (s, 1H), 11.24 (s, 1H), 7.98 (d, *J* = 8.0 Hz, 1H), 7.75 (t, *J* = 7.9 Hz, 1H), 7.40 (d, *J* = 8.4 Hz, 1H), 7.35 (t, *J* = 7.6 Hz, 1H). ^{13}C NMR (101 MHz, DMSO-d_6) δ 173.26, 157.07, 139.07, 135.67, 127.47, 124.78, 116.32, 116.19; LRMS: *m/z*(%)= 195.1 (100) $[M + H]^+$; HRMS: calcd. $C_8H_5O_2N_2S$ $[M - H]^-$: 193.0077; found: 193.0070;

Dose-response inhibition and data analysis

Approximate IC50 values were calculated (where possible) by analysing the inhibition of digestion of a radiolabelled RNA substrate. Gel images collected on a Typhoon scanner were analysed using Image J (NIH)7, determining the proportion of substrate remaining undigested (enzyme control) in comparison with the amount of that entered the gel determining the proportion of substrate remaining undigested (taking into account the no enzyme control); results are given as a percentage of digested substrate and were plotted against the log10 of the inhibitor concentration. The non-treated control was set to 0.3 μM when plotting the data on a semi-logarithmic scale, thus being less than one tenth of the first dose concentration (3.125 μM). Dose

response curves were fitted using nonlinear regression, and where possible IC50 values calculated. The data were plotted and IC50 values obtained in Graphpad Prism v8.3.0. Error is standard error.

Three or more gels were analysed for each inhibitor. Some data was excluded due to user determined factors such as: the quality of the gel being insufficient to obtain satisfactory data (e.g. gel cracked during drying), the signal of the radiation was below a minimum threshold, or the controls run on the gel were of unsatisfactory quality (e.g. bands running in a non-horizontal, or "smiling" format).

Differential scanning spectrometry (DSF)

DSF experiments were carried out using the method of Niesen *et al*[8]. Each of the compounds were serially diluted in DSF buffer (25 mM HEPES pH 7.5, 50 mM NaCl, 5 mM $MgCl_2$, 5% (v/v) glycerol, and 1 mM DTT). Note that DTT was excluded for experiments with thiram, disulfiram, and ebselen. The nsp14-10 complex (1 μM) was incubated in the presence of the compounds for 10 minutes at room temperature prior to the addition of SYPRO™ Orange protein stain (1:10000 dilution). The fluorescence emission was measured using a fluorescence resonance energy transfer filter (560–580 nm) with an excitation wavelength of 450–490 nm. During the DSF experiment, the temperature was increased from 25 to 95°C at an increment of 1°C per second.

REFERENCES

1 Savitsky, P. *et al.* High-throughput production of human proteins for crystallization: the SGC experience. *J Struct Biol* **172**, 3-13, doi:10.1016/j.jsb.2010.06.008 (2010).

2 Dominy, C. N. & Andrews, D. W. Site-directed mutagenesis by inverse PCR. *Methods Mol Biol* **235**, 209-223, doi:10.1385/1-59259-409-3:209 (2003).

3 Birch, A. J., Salahud-Din, M. & Smith, D. C. C. The synthesis of (±)-xanthorrhoein. *Journal of the Chemical Society C: Organic*, 523-527, doi:10.1039/J39660000523 (1966).

4 Falsini, M. *et al.* 3-Hydroxy-1H-quinazoline-2,4-dione as a New Scaffold To Develop Potent and Selective Inhibitors of the Tumor-Associated Carbonic

Anhydrases IX and XII. *J Med Chem* **60**, 6428-6439, doi:10.1021/acs.jmedchem.7b00766 (2017).

5 Tang, J. *et al.* 3-Hydroxypyrimidine-2,4-diones as an inhibitor scaffold of HIV integrase. *J Med Chem* **54**, 2282-2292, doi:10.1021/jm1014378 (2011).

6 Khokhlov, P. S., Osipov, V. N. & Roshchin, A. V. 3-Hydroxy- and 3-alkoxy-2-sulfanylquinazolin-4(3H)-ones: synthesis and reactions with alkylating and acylating agents. *Russian Chemical Bulletin* **60**, 153-156, doi:10.1007/s11172-011-0022-1 (2011).

7 Schneider, C. A., Rasband, W. S. & Eliceiri, K. W. NIH Image to ImageJ: 25 years of image analysis. *Nat Methods* **9**, 671-675, doi:10.1038/nmeth.2089 (2012).

8 Niesen, F. H., Berglund, H. & Vedadi, M. The use of differential scanning fluorimetry to detect ligand interactions that promote protein stability. *Nat Protoc* **2**, 2212-2221, doi:10.1038/nprot.2007.321 (2007).
