# Supplementary material for: Characterization of the SARS-CoV-2 ExoN (nsp14ExoN–nsp10) complex: implications for its role in viral genome stability and inhibitor identification
Source: Nucleic Acids Res. 2022 Jan 17;50(3):1484–500. doi: 10.1093/nar/gkab1303 (PMC8860572; doi:10.1093/nar/gkab1303)
Supplement: gkab1303_Supplemental_Files [file gkab1303_supplemental_files.zip › Baddock-Supplemental-data-combined.pdf]

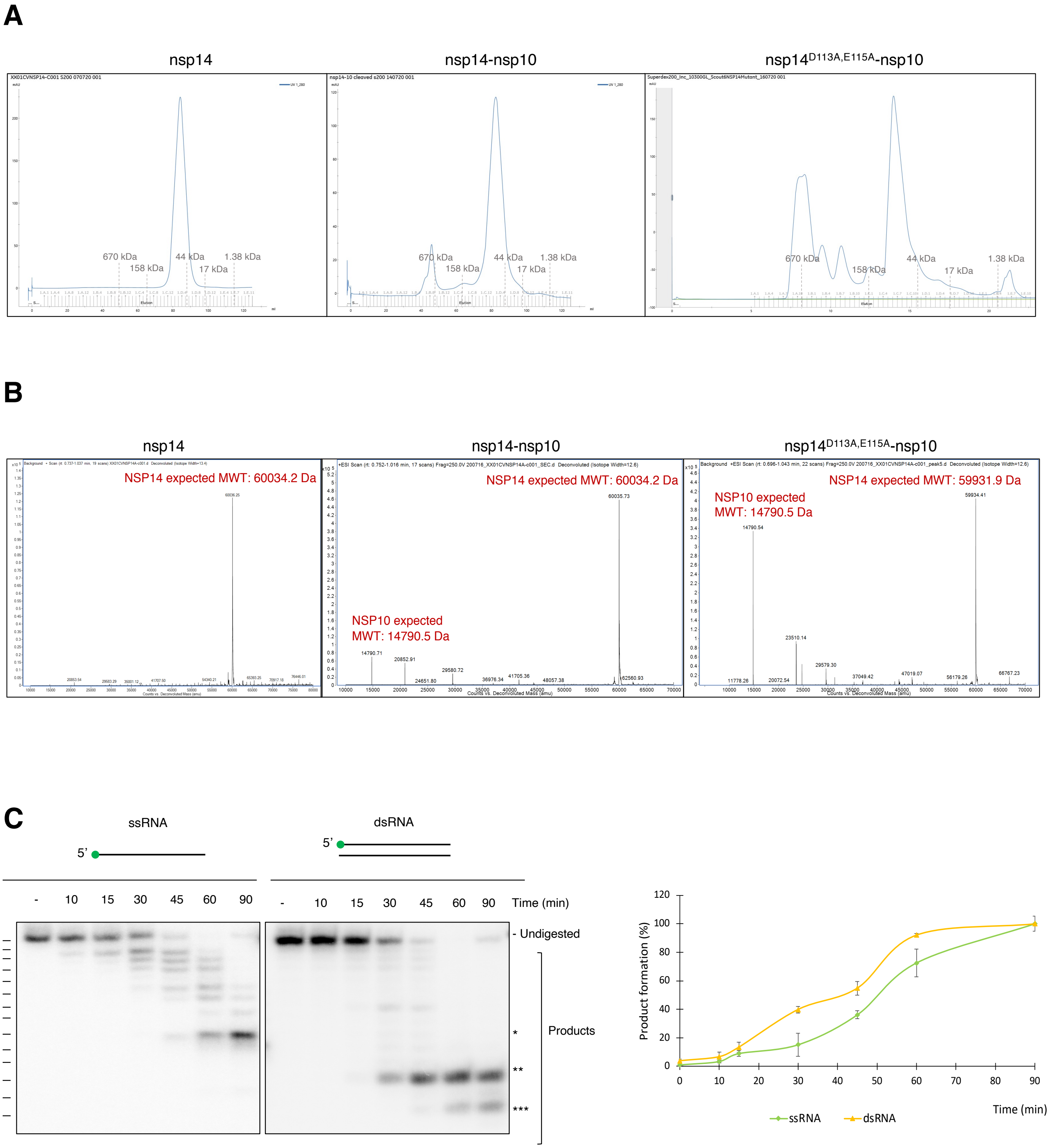

A
nsp14
nsp14-nsp10
nsp14D113A,E115A-nsp10
670 kDa
158 kDa
44 kDa
17 kDa
1.38 kDa
B
nsp14
NSP14 expected MWT: 60034.2 Da
nsp14-nsp10
NSP14 expected MWT: 60034.2 Da
NSP10 expected MWT: 14790.5 Da
nsp14D113A,E115A-nsp10
NSP14 expected MWT: 59931.9 Da
NSP10 expected MWT: 14790.5 Da
C
ssRNA
dsRNA
5'
- 10 15 30 45 60 90
- 10 15 30 45 60 90 Time (min)
20mer
19mer
12mer
10mer
7mer
- Undigested
Products
*
**
***
Product formation (%)
Time (min)
ssRNA
dsRNA

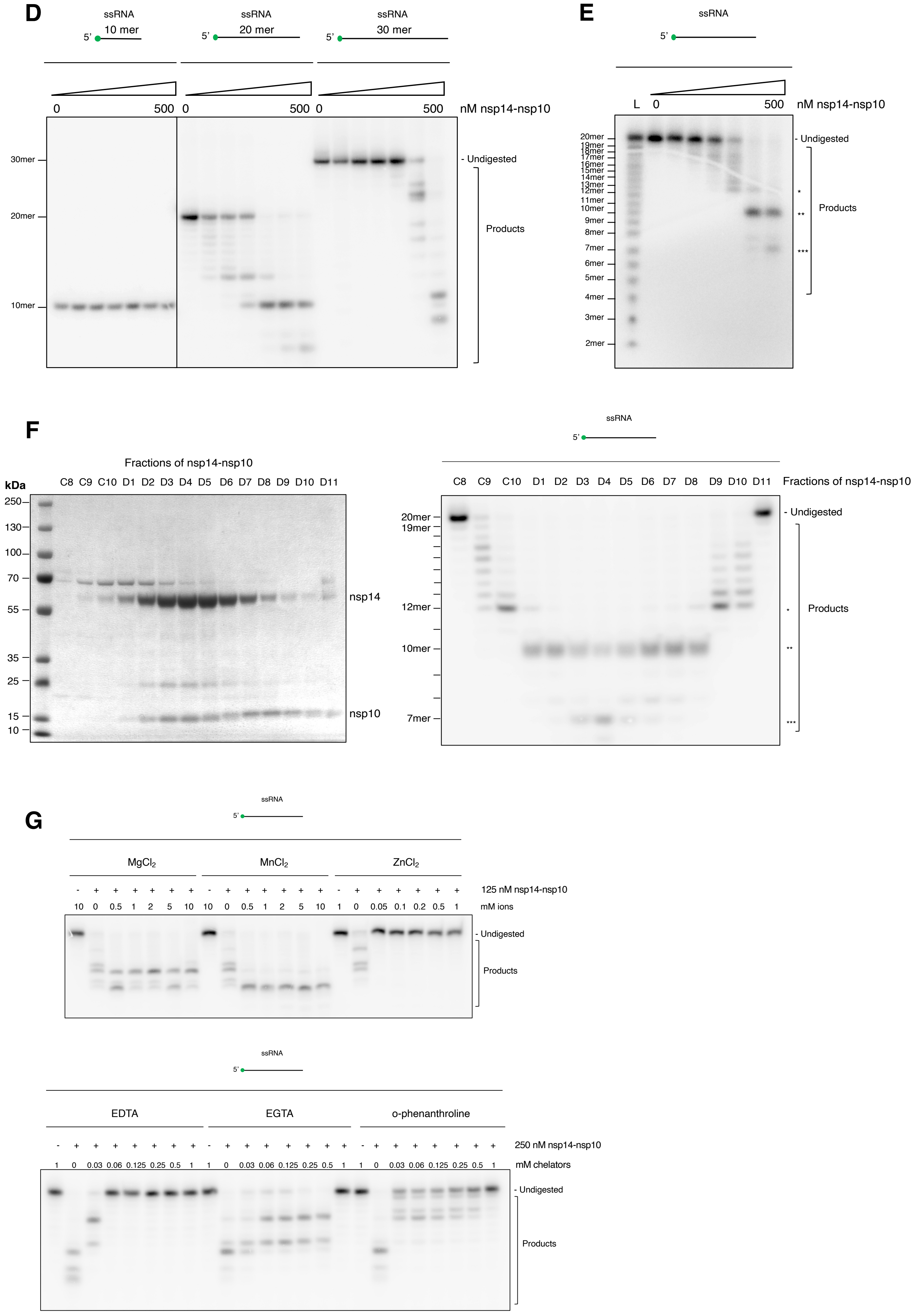
D
ssRNA
10 mer
5'
ssRNA
20 mer
5'
ssRNA
30 mer
5'
0
500
0
500
0
500
nM nsp14-nsp10
30mer
20mer
10mer
- Undigested
Products
E
ssRNA
5'
L
0
500
nM nsp14-nsp10
20mer
19mer
18mer
17mer
16mer
15mer
14mer
13mer
12mer
11mer
10mer
9mer
8mer
7mer
6mer
5mer
4mer
3mer
2mer
- Undigested
*
**
***
Products
F
Fractions of nsp14-nsp10
kDa
C8 C9 C10 D1 D2 D3 D4 D5 D6 D7 D8 D9 D10 D11
250
130
100
70
55
35
25
15
10
nsp14
nsp10
ssRNA
5'
C8 C9 C10 D1 D2 D3 D4 D5 D6 D7 D8 D9 D10 D11
Fractions of nsp14-nsp10
20mer
19mer
12mer
10mer
7mer
- Undigested
*
**
***
Products
G
ssRNA
5'
MgCl2
MnCl2
ZnCl2
- + + + + + + - + + + + + + - + + + + + +
125 nM nsp14-nsp10
10 0 0.5 1 2 5 10 10 0 0.5 1 2 5 10 1 0 0.05 0.1 0.2 0.5 1
mM ions
- Undigested
Products
ssRNA
5'
EDTA
EGTA
o-phenanthroline
- + + + + + + + - + + + + + + + - + + + + + + +
250 nM nsp14-nsp10
1 0 0.03 0.06 0.125 0.25 0.5 1 1 0 0.03 0.06 0.125 0.25 0.5 1 1 0 0.03 0.06 0.125 0.25 0.5 1
mM chelators
- Undigested
Products

Suppl. Figure 1: Nsp14-nsp10 is an RNA nuclease with a complex digestion pattern

A. Size exclusion chromatogram for nsp14 alone, wild-type nsp14-nsp10 (designated nsp14-nsp10), and a control 'nuclease-dead' complex bearing alanine substitutions at residues D113 and E115 (nsp14^{D113A,E115A}-nsp10). Traces for Superdex 200 16/60 chromatography are shown for Nsp14 alone and wild-type nsp14-nsp10 and trace of Superdex 10/300 run for the 'nuclease-dead' complex. Wild-type nsp14-nsp10 complex elutes in a single peak at ~82.5 mL, Nsp14 alone at ~84.3 mL and 'nuclease-dead' complex at ~14.6 mL . Molecular weight standards were run on all columns and are in grey. SDS-PAGE analysis of the peak shows the presence of both nsp14 and nsp10 in fractions D1–D8 (see Suppl. Fig. 1e).

B. Intact mass spectrometry analysis of nsp14 alone, wild-type nsp14-nsp10 (designated nsp14-nsp10) and a control 'nuclease-dead' complex bearing substitutions at residues D113 and E115 (nsp14^{D113A,E115A}-nsp10). LC-MS analysis shows the observed mass of all proteins is within range of the calculated mass.

C. Time course assays containing 30 nM nsp14-nsp10 for ssRNA and 60 nM nsp14-nsp10 for dsRNA were used to define the linear range of product formation. Product formation (%) comparing nsp14-nsp10 nuclease activity on ssRNA and dsRNA over time was quantified as outlined in Methods and Materials. All data are shown as mean ± s.e.m. Oligonucleotides 2 and 3 were used.

D. Nsp14-nsp10 is able to digest a 20-mer and a 30-mer ssRNA substrate, but not a 10-mer ssRNA. Oligos 1, 2 and 17 were used respectively.

E. Nsp14-nsp10 is an RNA nuclease with a complex digestion pattern, digesting from the 3' end in a single-nucleotide fashion until the 8th ribonucleotide, then cleaving at the 10th and 13th ribonucleotide. A single nucleotide ladder was used to determine the size of the released products.

F. SDS-PAGE of the fractions before, during and after the peak used from gel filtration for wild-type nsp14-nsp10. The elution peak (D1–D8) coincides precisely with the characteristic RNAse activity that we observe in our activity gel. The predicted molecular weight is 59 463 Da for nsp14 and 15 281 Da for nsp10.

G. Nsp14-nsp10 activity is enhanced by the addition of both $MgCl_2$ and $MnCl_2$ but is inhibited by $ZnCl_2$. We determined 5 mM $MgCl_2$ as optimal for activity under the tested conditions. Consistent with its requirement for metal ions, nsp14-nsp10 activity is inhibited by the addition of metal ion chelators EDTA, EGTA, and *o*-phenanthroline, with a particular sensitivity towards EDTA.

   Increasing concentrations of protein (as indicated) were incubated with substrate at 37°C for 45 min, reactions were subsequently analysed by 20% denaturing PAGE. For ions and chelators gels, 125 nM or 250 nM of protein was incubated with increasing concentrations of ions or chelators (as indicated) on ice for 15 min before adding substrate and incubating the reaction at 37°C for 45 min. Reactions were subsequently analysed by 20% denaturing PAGE.The size of products was determined as shown in Suppl. Fig 1E. Main products are labelled *, ** and *** corresponding to 12-mer, 10-mer and 7-mer respectively. All oligos used are indicated in Suppl. table 1A and B. For analyses shown in Suppl. Figs. 1E, F and G, oligonucleotide 2 was used.

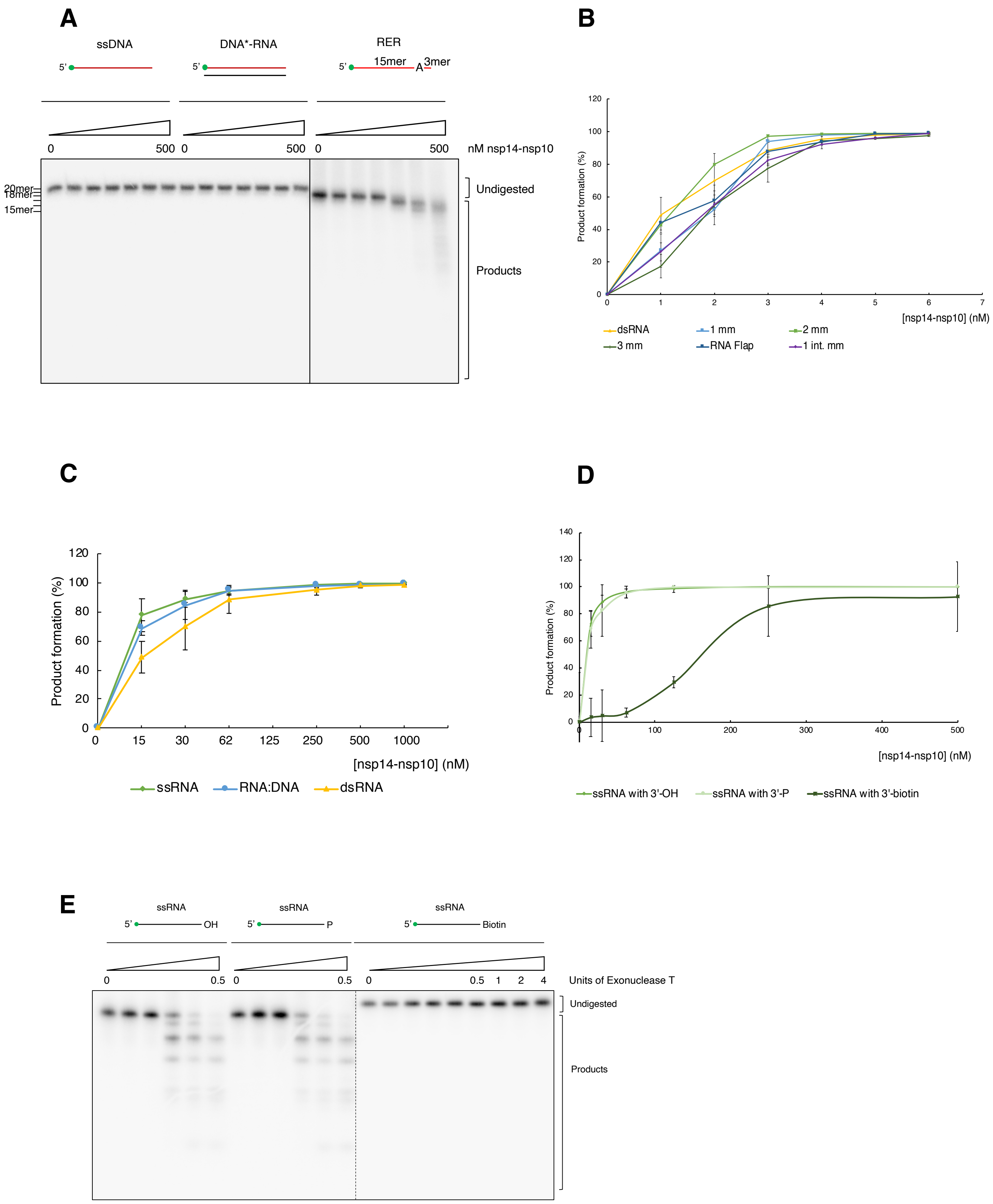

A
ssDNA
DNA*-RNA
RER
5'
15mer
A
3mer
0
500
nM nsp14-nsp10
20mer
18mer
15mer
Undigested
Products
B
Product formation (%)
[nsp14-nsp10] (nM)
dsRNA
1 mm
2 mm
3 mm
RNA Flap
1 int. mm
C
Product formation (%)
0
15
30
62
125
250
500
1000
[nsp14-nsp10] (nM)
ssRNA
RNA:DNA
dsRNA
D
Product formation (%)
[nsp14-nsp10] (nM)
ssRNA with 3'-OH
ssRNA with 3'-P
ssRNA with 3'-biotin
E
ssRNA
5'
OH
P
Biotin
0
0.5
1
2
4
Units of Exonuclease T
Undigested
Products

F

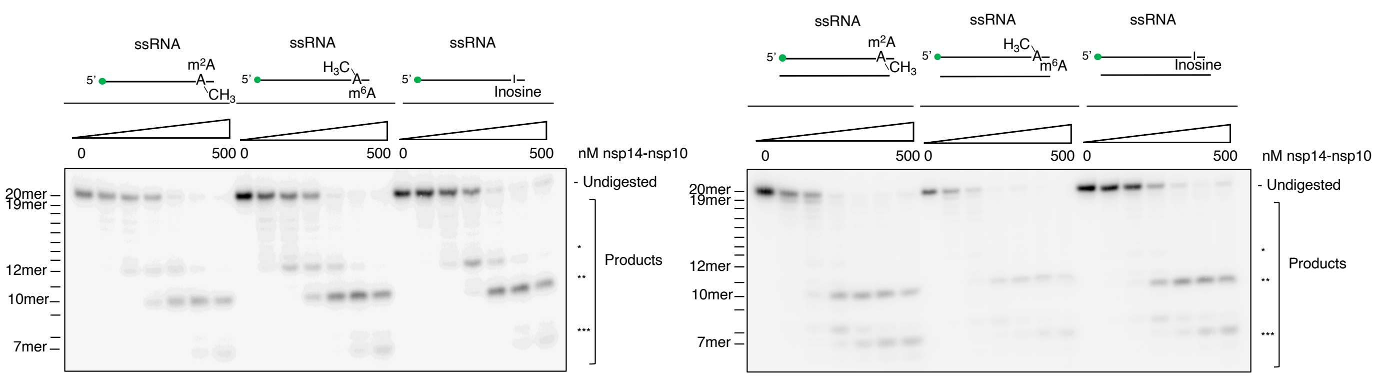
ssRNA
m2A
CH3
ssRNA
H3C
m6A
ssRNA
Inosine
0
500
nM nsp14-nsp10
20mer
19mer
12mer
10mer
7mer
- Undigested
Products
*
**
***

G

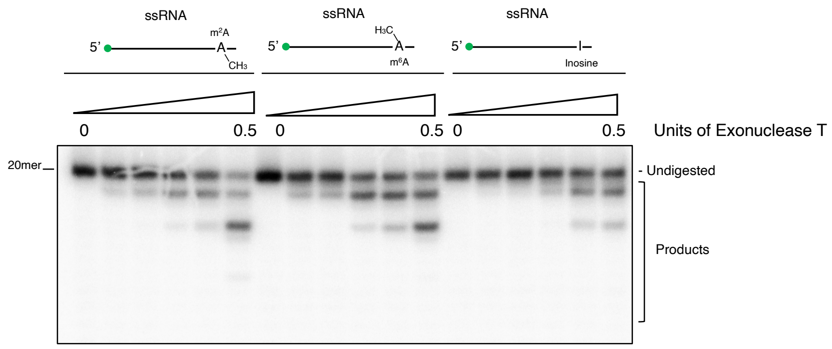
ssRNA
m2A
CH3
ssRNA
H3C
m6A
ssRNA
Inosine
0
0.5
Units of Exonuclease T
20mer
- Undigested
Products

Suppl. Figure 2: Nsp14-nsp10 activity is specific to RNA only and is able to process common chemical modifications on RNA

A. Nsp14-nsp10 has no discernible nuclease activity on ssDNA or the DNA strand of an DNA:RNA hybrid. Interestingly, the complex is able to incise around an embedded ribonucleotide in a ssDNA oligo at concentrations above 125 nM.

B. Product formation (%) was quantified for Fig. 1 C comparing nsp14-nsp10 nuclease activity on dsRNA and RNA substrates containing termini and internal mismatches as outlined in Methods and Materials. All data are shown as mean ± s.e.m, and at least three biological replicates were used for each substrate. Nsp14-nsp10 shows no preference for mismatched oligonucleotides compared to dsRNA.
mm: mismatch
int. mm: internal mismatch

C. Product formation (%) was quantified for Fig. 2 C comparing nsp14-nsp10 nuclease activity on ssRNA, the RNA strand of an RNA:DNA hybrid and dsRNA as outlined in Methods and Materials. All data are shown as mean ± s.e.m, and at least three biological replicates were used for each substrate. Nsp14-nsp10 shows equivalent activity on ssRNA and the RNA strand of an RNA:DNA hybrid with a slight decrease in activity for dsRNA.

D. Product formation (%) was quantified for Fig. 2 D comparing nsp14-nsp10 nuclease activity on ssRNA with a 3'-hydroxyl, a 3'-phosphate or a 3'-biotin group as outlined in Methods and Materials. All data are shown as mean ± s.e.m, and at least three biological replicates were used for each substrate. Nsp14-nsp10 shows equivalent activity a ssRNA with a 3'-hydroxyl and a 3'-phosphate but reduced activity on ssRNA with a 3'-biotin group.

E. Exonuclease T was used to demonstrate a classic RNA exonuclease activity. Increasing concentrations of protein (0 to 0.5 U) were incubated with substrate at 30°C for 45 min and reactions were subsequently analysed by 20% denaturing PAGE. With a substrate containing a 3'-biotin group, no activity is observed (even at 4 U) confirming ExoT has no endonuclease activity while the substrates with a 3'- hydroxyl or phosphate exhibit nearly identical product formation. Oligos 2, 15 and 16 were used respectively (see Suppl. Table 1A).

F. Nsp14-nsp10 is able to process common chemical modifications of RNA, namely 2-methyladenine (m^2A), 6-methyladenine (m^6A) and inosine (I), both ssRNA and dsRNA with no apparent change compared to ssRNA or dsRNA.

G. Exonuclease T is also able to process common chemical modifications of RNA, namely 2-methyladenine (m^2A), 6-methyladenine (m^6A) and inosine (I).

   Increasing concentrations of protein (as indicated) were incubated with substrate at 37°C for 45 min, reactions were subsequently analysed by 20% denaturing PAGE. The size of products was determined as shown in Suppl. Fig 1E. Main products are labelled *, ** and *** corresponding to 12-mer, 10-mer and 7-mer respectively. Oligomer substrates are given in Suppl. Table 1A and B.

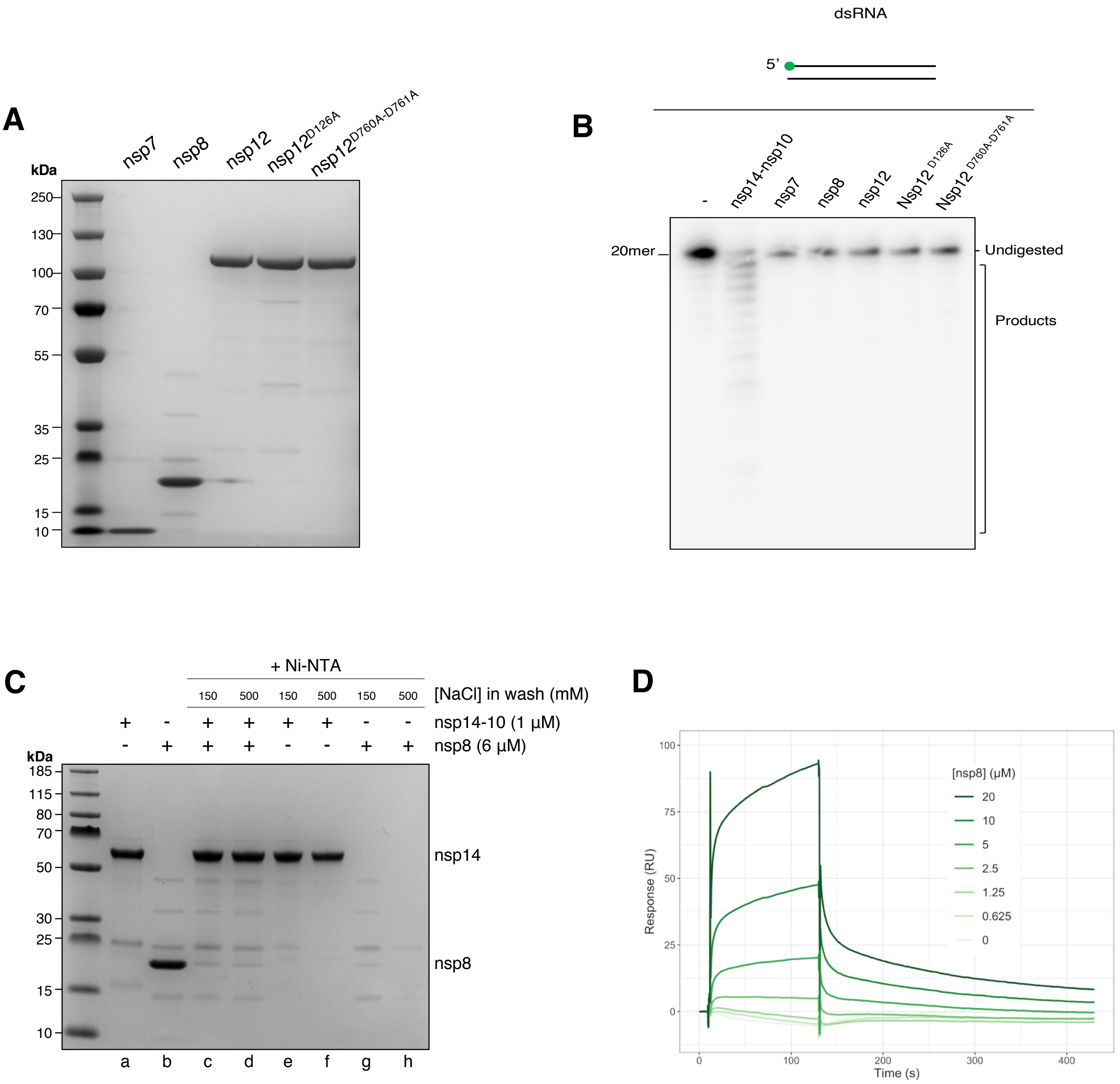
A
kDa
nsp7
nsp8
nsp12
nsp12D126A
nsp12D760A-D761A
250
130
100
70
55
35
25
15
10
B
dsRNA
5'
-
nsp14-nsp10
nsp7
nsp8
nsp12
Nsp12 D126A
Nsp12 D760A-D761A
20mer
Undigested
Products
C
+ Ni-NTA
150
500
150
500
150
500
[NaCl] in wash (mM)
nsp14-10 (1 µM)
nsp8 (6 µM)
kDa
185
115
80
70
50
30
25
15
10
nsp14
nsp8
a
b
c
d
e
f
g
h
D
Response (RU)
Time (s)
[nsp8] (µM)
20
10
5
2.5
1.25
0.625
0
100
75
50
25
0
100
200
300
400

Suppl. Figure 3: Investigating the stimulation of SARS-CoV-2 nsp14-nsp10 by nsp12-7-8 polymerase complex

A. SDS-PAGE of purified nsp7, nsp8, np12, nsp12^{D126A} and nsp12$^{D760A-D761A}$ showing the purity of the purified proteins. Predicted molecular weights are 9 000 Da for nsp7, 22 000 Da for nsp8, 106 000 Da for nsp12, nsp12^{D126A} and nsp12$^{D760A-D761A}$.

B. All components of the SARS-CoV-2 polymerase complex used in this study (nsp7, nsp8, nsp12 as well as catalytic mutants nsp12^{D126A} and nsp12$^{D760A-D761A}$; 500nM) were incubated with substrate at 37°C for 45 min, reactions were subsequently analysed by 20% denaturing PAGE to visualize product formation. No digestion of the RNA substrate was observed. Nsp14-nsp10 (100nM) was used as a positive control. All oligos used are indicated in Suppl. table 1A and B.

C. A weak interaction was observed between the nuclease nsp14 and the primase nsp8. Ni-NTA pulldown of purified nsp14-nsp10 (as bait), with nsp8. Bound proteins were washed with either low (150 mM) NaCl or low + high (500 mM) NaCl. All proteins were visualised with SDS PAGE, with comparisons to input proteins in lanes a–b. Lanes c and d show evidence for interaction with nsp8 when nsp14-nsp10 was used as a bait.

D. Using a Biacore SPR system, a weak interaction between nsp14-nsp10 and nsp8 was identified by immobilising nsp14-nsp10 onto a CM5 chip using an amide coupling kit, while running varying concentrations of nsp8 through the cell.

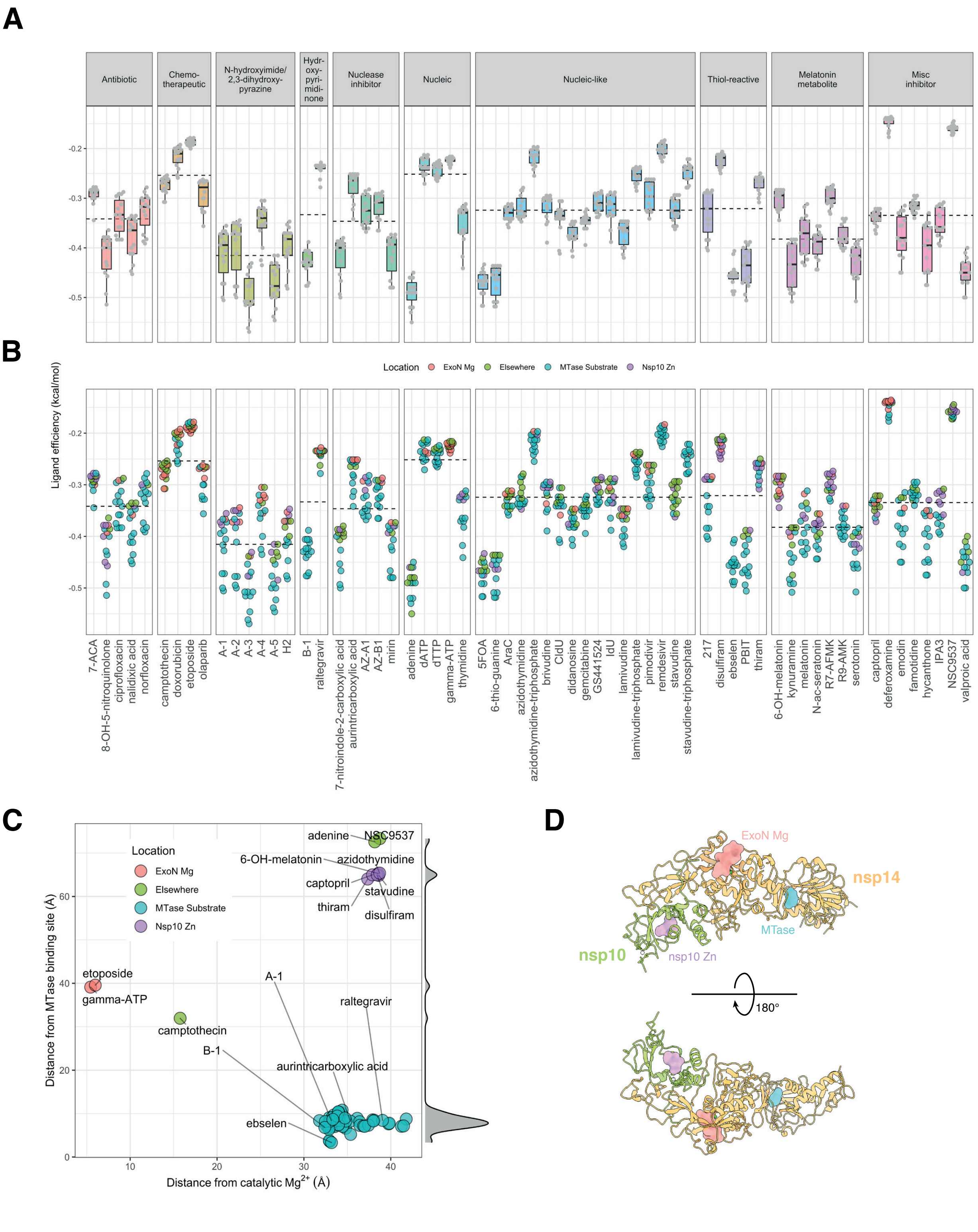
A
Antibiotic
Chemo-therapeutic
N-hydroxyimide/ 2,3-dihydroxy-pyrazine
Hydr-oxy-pyri-midi-none
Nuclease inhibitor
Nucleic
Nucleic-like
Thiol-reactive
Melatonin metabolite
Misc inhibitor
-0.2
-0.3
-0.4
-0.5
B
Ligand efficiency (kcal/mol)
Location
ExoN Mg
Elsewhere
MTase Substrate
Nsp10 Zn
7-ACA
8-OH-5-nitroquinolone
ciprofloxacin
nalidixic acid
norfloxacin
camptothecin
doxorubicin
etoposide
olaparib
A-1
A-2
A-3
A-4
A-5
H2
B-1
raltegravir
7-nitroindole-2-carboxylic acid
aurintricarboxylic acid
AZ-A1
AZ-B1
mirin
adenine
dATP
dTTP
gamma-ATP
thymidine
5FOA
6-thio-guanine
AraC
azidothymidine
azidothymidine-triphosphate
brivudine
CldU
didanosine
gemcitabine
GS441524
IdU
lamivudine
lamivudine-triphosphate
pimodivir
remdesivir
stavudine
stavudine-triphosphate
217
disulfiram
ebselen
PBIT
thiram
6-OH-melatonin
kynuramine
melatonin
N-ac-seratonin
R7-AFMK
R9-AMK
serotonin
captopril
deferoxamine
emodin
famotidine
hycanthone
IPA3
NSC9537
valproic acid
C
Location
ExoN Mg
Elsewhere
MTase Substrate
Nsp10 Zn
adenine
NSC9537
6-OH-melatonin
azidothymidine
captopril
stavudine
thiram
disulfiram
etoposide
gamma-ATP
camptothecin
A-1
raltegravir
B-1
aurintricarboxylic acid
ebselen
Distance from MTase binding site (Å)
Distance from catalytic Mg2+ (Å)
0
20
40
60
10
20
30
40
D
ExoN Mg
nsp14
MTase
nsp10
nsp10 Zn
180°

Suppl. Figure 4: Molecular docking of SARS-CoV nsp14-nsp10 using Autodock

A. Nsp14-nsp10 was docked with compounds within a grid box encompassing a surface focussed on the active site. The calculated affinities of all binding modes calculated are shown for each compound, grouped based on functionality of the compound; a dashed line describes the median of affinities in a set.

B. Nsp14-nsp10 was docked with compounds within a grid box encompassing the surface and affinities of all binding modes calculated are shown for each compound, grouped based on *in vitro* inhibiting activity and coloured based on location of binding mode on the structure of nsp14-nsp10; dashed line describes the median of affinities in this cohort.

C. The positions of highest-affinity docked poses are summarised as the distance from the active site ExoN Mg^{2+} centre against distance from the MTase substrate (GpppA) binding site.

D. Three representative locations are shown on the structure of nsp14-nsp10; purple -nsp10 Zn^{2+} proximal (disulfiram), salmon - ExoN Mg^{2+} proximal (etoposide), cyan - MTase substrate binding site (A-1).

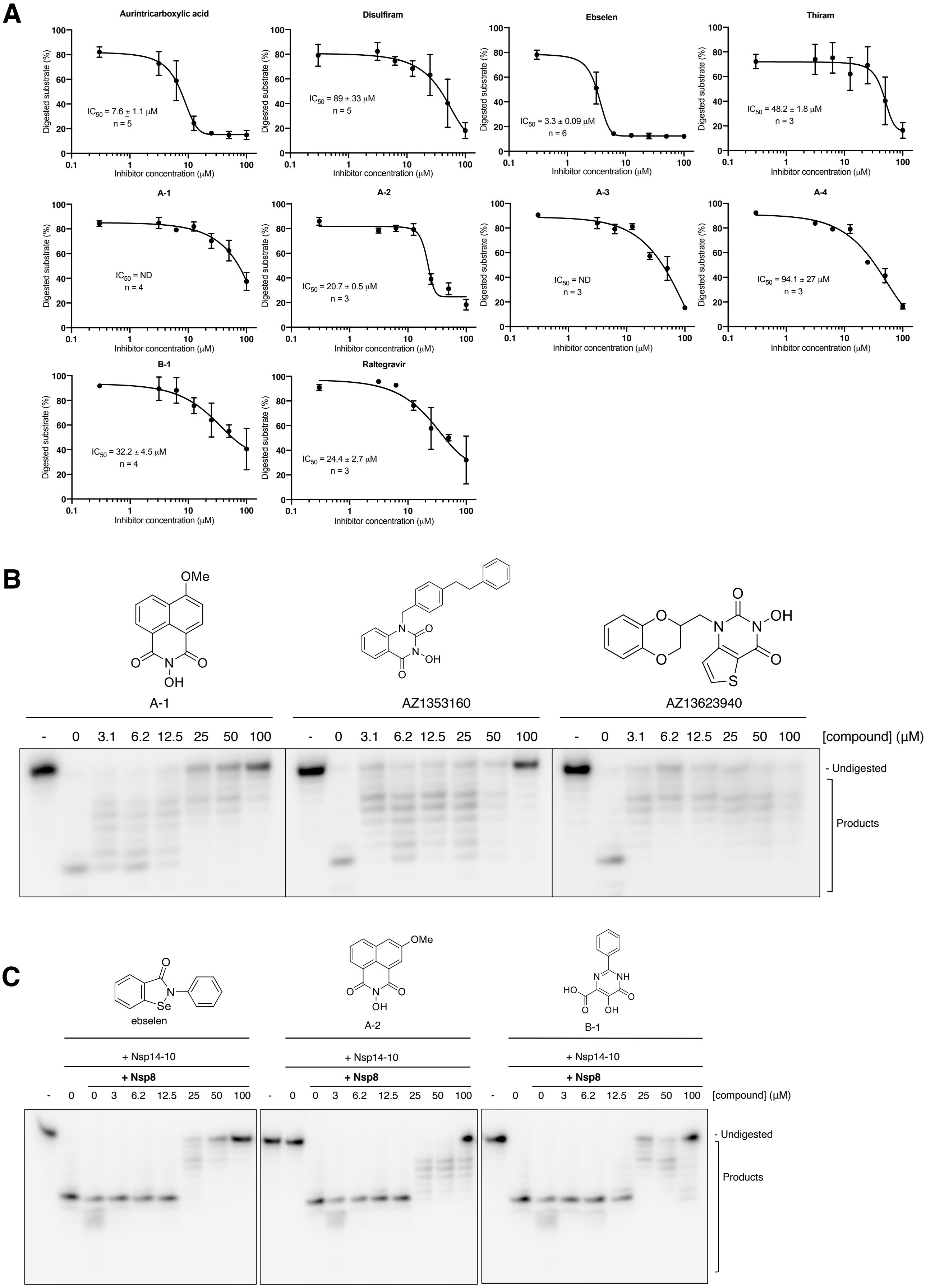

A
Aurintricarboxylic acid
IC50 = 7.6 ± 1.1 μM
n = 5
Disulfiram
IC50 = 89 ± 33 μM
n = 5
Ebselen
IC50 = 3.3 ± 0.09 μM
n = 6
Thiram
IC50 = 48.2 ± 1.8 μM
n = 3
A-1
IC50 = ND
n = 4
A-2
IC50 = 20.7 ± 0.5 μM
n = 3
A-3
IC50 = ND
n = 3
A-4
IC50 = 94.1 ± 27 μM
n = 3
B-1
IC50 = 32.2 ± 4.5 μM
n = 4
Raltegravir
IC50 = 24.4 ± 2.7 μM
n = 3
Digested substrate (%)
Inhibitor concentration (μM)
B
OMe
O
N
OH
A-1
AZ1353160
AZ13623940
- 0 3.1 6.2 12.5 25 50 100
[compound] (μM)
- Undigested
Products
C
ebselen
A-2
B-1
+ Nsp14-10
+ Nsp8
- 0 0 3 6.2 12.5 25 50 100
[compound] (μM)
- Undigested
Products

D

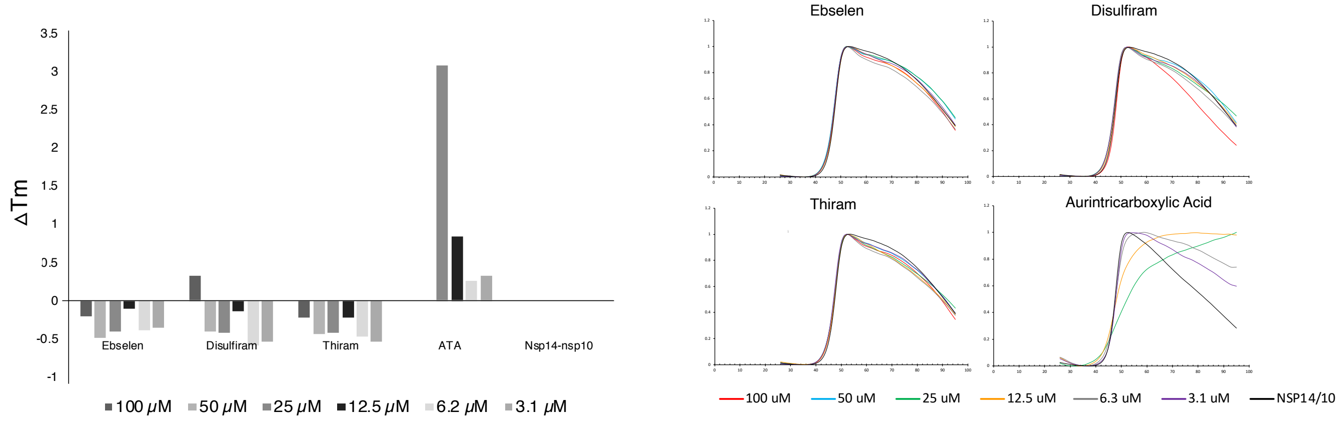
ΔTm
3.5
3
2.5
2
1.5
1
0.5
0
-0.5
-1
Ebselen
Disulfiram
Thiram
ATA
Nsp14-nsp10
100 μM
50 μM
25 μM
12.5 μM
6.2 μM
3.1 μM
Ebselen
Disulfiram
Thiram
Aurintricarboxylic Acid
100 uM
50 uM
25 uM
12.5 uM
6.3 uM
3.1 uM
NSP14/10

E

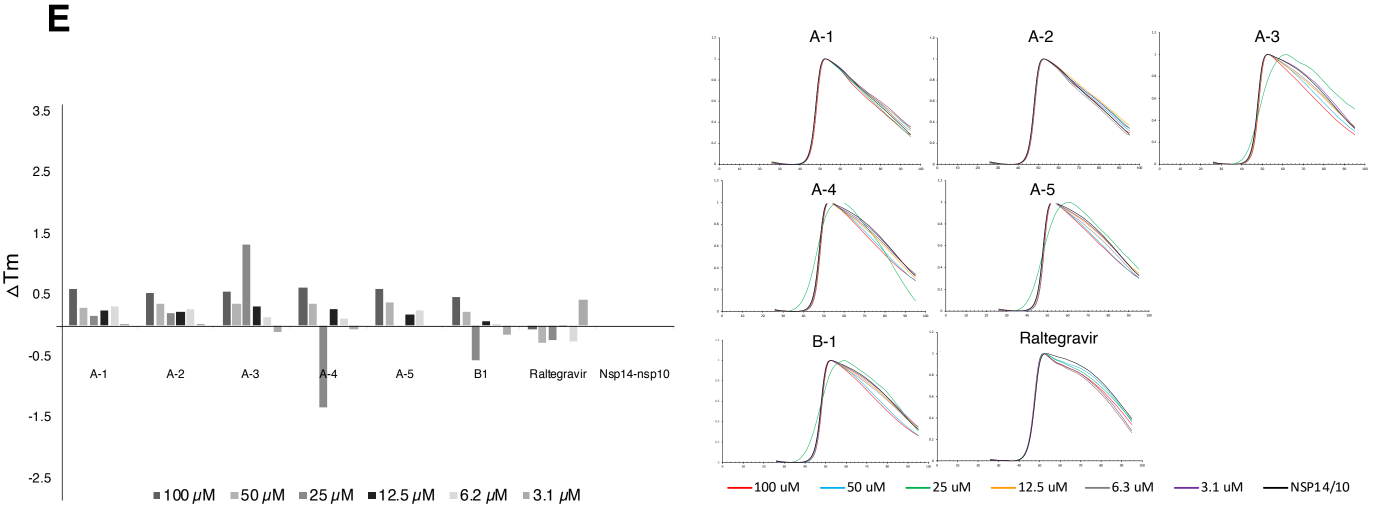
ΔTm
3.5
2.5
1.5
0.5
-0.5
-1.5
-2.5
A-1
A-2
A-3
A-4
A-5
B1
Raltegravir
Nsp14-nsp10
100 μM
50 μM
25 μM
12.5 μM
6.2 μM
3.1 μM
A-1
A-2
A-3
A-4
A-5
B-1
Raltegravir
100 uM
50 uM
25 uM
12.5 uM
6.3 uM
3.1 uM
NSP14/10

F

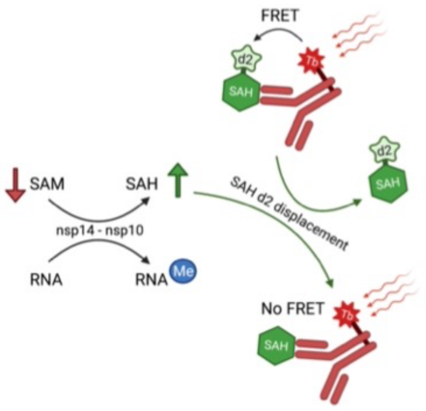
FRET
d2
SAH
Tb
SAM
SAH
nsp14 - nsp10
RNA
RNA
Me
SAH d2 displacement
d2
SAH
No FRET
SAH
Tb

G

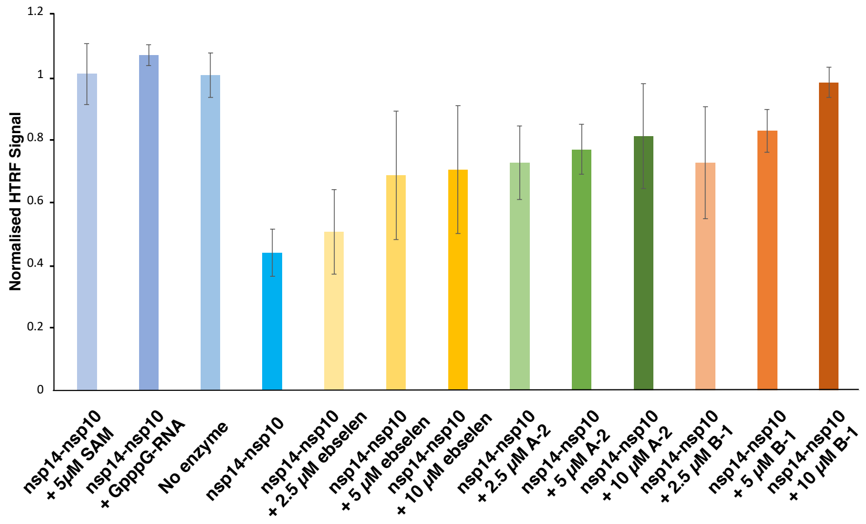
Normalised HTRF Signal
1.2
1
0.8
0.6
0.4
0.2
0
nsp14-nsp10 + 5μM SAM
nsp14-nsp10 + GpppG-RNA
No enzyme
nsp14-nsp10
nsp14-nsp10 + 2.5 μM ebselen
nsp14-nsp10 + 5 μM ebselen
nsp14-nsp10 + 10 μM ebselen
nsp14-nsp10 + 2.5 μM A-2
nsp14-nsp10 + 5 μM A-2
nsp14-nsp10 + 10 μM A-2
nsp14-nsp10 + 2.5 μM B-1
nsp14-nsp10 + 5 μM B-1
nsp14-nsp10 + 10 μM B-1

Suppl. Figure 5: Nsp14-nsp10 inhibitor analysis

A. Inhibition profile curves were calculated by quantification of gel-based nuclease assay data as outlined in Methods and Materials. A decrease in the '% digested' of the substrate represents an increase in inhibition. Data were plotted against inhibitor concentration on a semi-logarithmic scale and dose response curves were generated using non-linear regression. Where possible IC_{50} values were obtained using 100 nM nsp14-nsp10. All data are shown as mean ± s.e.m, and at least three biological replicates were used for each compound. In some cases, an IC_{50} value was unable to be calculated, either due to weak inhibition, or the inability to fit a sigmoidal curve to the data. In some cases, the precise IC_{50} values should be interpreted cautiously due to the nature of the curves obtained.

B. Increasing concentrations (as indicated, in µM) of A-1, AZ1353160 (AZ-A1) and AZ13623940 (AZ-B1) were incubated with 100 nM nsp14-nsp10 for 10 minutes at room temperature, before starting a standard nuclease assay reaction by the addition of ssRNA, incubating at 37°C for 45 min. Reaction products were analysed by 20% denaturing PAGE. A decrease in the generation of nucleolytic reaction products and a concomitant increase in undigested substrate indicates inhibition of nuclease activity at increasing inhibitor concentrations. - indicates no enzyme.

C. Increasing concentrations (as indicated, in µM) of ebselen, A-2 and B-1 were incubated with 100 nM nsp14-nsp10 and 300 nM nsp8 for 10 minutes at room temperature, before starting a standard nuclease assay reaction by the addition of ssRNA, incubating at 37°C for 45 min. Reaction products were analysed by 20% denaturing PAGE. A decrease in the generation of nucleolytic reaction products and a concomitant increase in undigested substrate indicates inhibition of nuclease activity at increasing inhibitor concentrations. - indicates no enzyme.

D. Differential scanning spectrometry (DSF) of nsp14-nsp10 with drug and drug-like compounds and *N*-hydroxyimide and hydroxypyrimidinone based compounds. ΔTm of nsp14-nsp10 with drugs and drug-like compounds. Of all the compounds tested, only aurintricarboxylic acid (ATA), a known pan-nuclease inhibitor appears to aggregate the protein at high concentration (100 μM and 50 μM).

E. Differential scanning spectrometry (DSF) of nsp14-nsp10 with *N*-hydroxyimide and hydroxypyrimidinone based compounds. ΔTm of nsp14-nsp10 with *N*-hydroxyimide compounds. Samples were heated from 25–95°C in 1°C per minute increments. Fluorescence intensities were plotted as a function of temperature to generate a sigmoidal curve with the inflection point of the transition curve indicating the melting temperature (Tm) of the protein. The Tm of nsp14-nsp10 is around 47°C. The ΔTm for each of the compound dilutions are shown as inset table. See Suppl. Materials and Methods for more detail.

F. Outline of the HTRF based assay for methyltransferase activity. nsp14-nsp10 is a SAM-dependent methyltransferase that produces SAH following methyltransfer to their substrate. SAH displaces SAH-d2 from the variable region of an α-SAH Tb cryptate-conjugated antibody, lowering HTRF signal through the disruption of the Tb cryptate – d2 FRET pair.

G. nsp14-nsp10 was assayed for methyltransferase activity through the HTRF based assay. The methyltransferase reaction was run with 10 nM nsp14-nsp10, 5 µM SAM, 0.14 mM GP_3G-RNA or different combinations of these three components. The methyltransferase reaction was conducted in the presence of 2.5 µM, 5 µM and 10 µM of ebselen, A-2 and B-1 identified. In all cases, nsp14-nsp10 methyltransferase activity was inhibited, albeit differentially.

A

NAME	DESCRIPTION	SEQUENCE (5' to 3')	Minimum Free Energy (kcal/mol)
1	10mer RNA	AUA AUU AGA U	0
2	20mer RNA	AUA AUU UGA UCA UCU AUU AU	0
3	20mer RNA	AUA AUA GAU GAU CAA AUU AU	0
4	20mer RNA	AUC UAA ACG AAC AAA CUA AA	0
5	20mer RNA	CAA CAG UUC AAG AAA UCA AA	0
6	20mer RNA	AUA AUA GAU GAU CAA AUU AG	0
7	20mer RNA	AUA AUA GAU GAU CAA AUU CG	0
8	20mer RNA	AUA AUA GAU GAU CAA AUG CG	0
9	20mer RNA	AUA AUA GAU GAU CAA ACG CG	0
10	20mer RNA	AUA AUA GAU GAC CAA AUU AU	0
11	20mer RNA	UUU UUU UUU UUU UUU UUU UU	0
12	20mer RNA	AAA AAA AAA AAA AAA AAA AA	0
13	20mer RNA	CCC CCC CCC CCC CCC CCC CC	0
14	20mer RNA	GGG GGG GGG GGG GGG GGG GG	0
15	20mer RNA with 3'-Phosphate	AUA AUU UGA UCA UCU AUU AU [PHO]	0
16	20mer RNA with 3'- Biotin	AUA AUU UGA UCA UCU AUU AU [BIOTEG]	0
17	30mer RNA	GUU GAG AGA GAG AGA GUU UGG AGA GUU UGU	0
18	20mer DNA	ATA ATA GAT GAT CAA ATT AT	0
19	18mer DNA with a single ribonucleotide at position 15	GAT CTG AGC CTG GG[A] GCT	-3.3
20	20mer RNA with a 2-methyladenosine at position 19	AUA AUU UGA UCA UCU AUU A*U	0
21	20mer RNA with a N6-methyladenosine at position 19	AUA AUU UGA UCA UCU AUU A*U	0
22	20mer RNA with an inosine at position 19	AUA AUU UGA UCA UCU AUU I*U	0
23	20mer RNA	GUC AUU CUC CUA AGA AGC UA	-0.3

B

STRUCTURE	CODE
RNA-DNA hybrid	2 + 18
double-stranded RNA	2 + 3
double-stranded RNA with a 2-methyladenosine on the top strand	20 + 3
double-stranded RNA with a N6-methyladenosine on the top strand	21 + 3
double-stranded RNA with a inosine on the top strand	22 + 3
double-stranded RNA with one terminal mismatch	6 + 2
double-stranded RNA with two terminal mismatches	7 + 2
double-stranded RNA with three terminal mismatches	8 + 2
RNA Flap	9 + 2
double-stranded RNA with one internal mismatch at position 12	10 + 2

Suppl. Table 1: List of RNA and DNA oligonucleotide sequences used to generate simple and complex RNA and DNA-containing substrates

A. Each oligonucleotide sequence is numbered and a general description given. Minimum free energy were calculated using RNAFold 2.4.7 as part of the ViennaRNA suite (1).

B. To generate complex RNA and DNA structures, single-stranded oligos were annealed as designated by the numbers using the protocol described in the Materials and Methods.

Compound	Structure	Function	FDA approval	Level of inhibition	AutoDock Vina Full Surface (kcal/mol)	AutoDock Vina Active Site (kcal/mol)
7-nitroindole-2-carboxylic acid (CRT0044876)	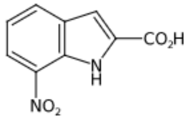	Nuclease inhibitor (APE1)	No.	Some inhibition.	-7.5	-6.2
AraC (cytarabine)	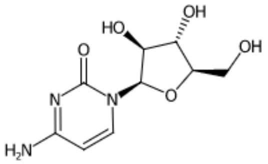	Nucleoside analogue	Yes (chemotherapeutic; treatment of acute myeloid leukaemia, acute lymphocytic leukaemia, lymphoma).	No inhibition.	-6.1	-5.7
aurintricarboxylic acid	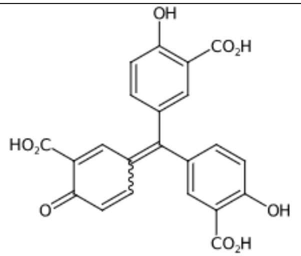	RNase inhibitor	No.	Yes, IC_{50} = 7.4 μM.	-10.6	-7.5
brivudine	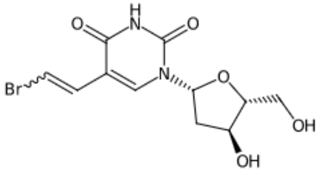	Nucleoside analogue	Yes (antiviral; treatment of herpes zoster).	No inhibition.	-7.4	-6.4

ciprofloxacin	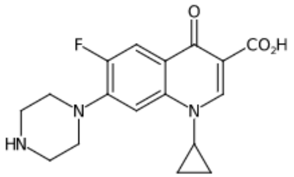	Antibiotic	Yes (quinolone antibiotic; broad antibacterial usage).	Incomplete inhibition, IC_{50} unable to be calculated.	-9.2	-6.6
didanosine	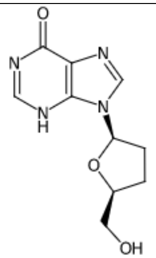	Nucleoside analogue	Yes (reverse transcriptase inhibitor; management of HIV).	No inhibition.	-7.1	-5.8
doxorubicin	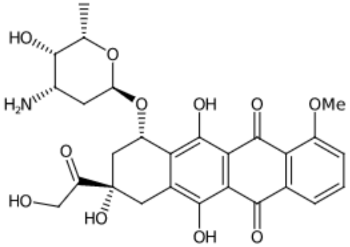	Topoisomerase inhibitor	Yes (chemotherapeutic; broadly used, including, breast cancer, bladder cancer, lymphoma, and acute lymphocytic leukaemia).	Some inhibition.	-10.1	-8.0
ebselen	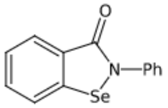	Thiol-reactive	In phase III clinical trials (bipolar disorder, hearing loss, sensory disorders).	Yes, IC_{50} = 3.3 μM.	-7.9	-5.9
emodin	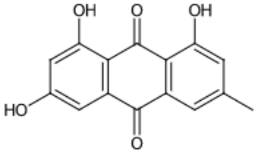	Quinone	No, but is available as a dietary supplement.	No inhibition.	-9.0	-7.3

famotidine	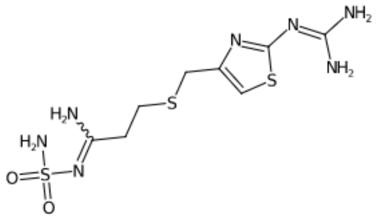	H2 receptor antagonist	Yes (antacid; treatment of peptic ulcer disease, gastroesophageal reflux disease).	No inhibition.	-6.8	-5.6
gemcitabine	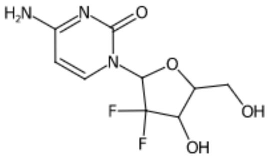	Nucleoside analogue	Yes (chemotherapeutic; broadly used, including, breast cancer, ovarian cancer, non-small cell lung cancer).	No inhibition.	-7.1	-5.5
lamivudine	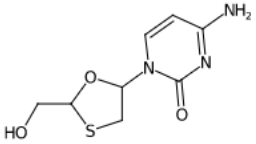	Nucleoside analogue	Yes (reverse transcriptase inhibitor; management of HIV).	No inhibition.	-6.3	-5.4
melatonin	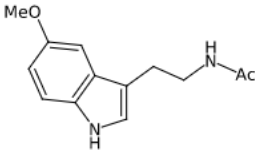	Tryptophan derivative (hormone)	No, but available as a dietary supplement, and is a natural metabolite.	Some inhibition.	-7.4	-5.9
pimodivir	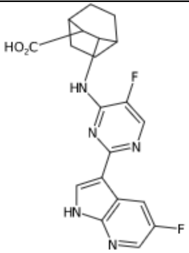	Polymerase inhibitor	In phase II clinical trials (treatment of influenza virus).	No inhibition.	-9.9	-8.1

remdesivir	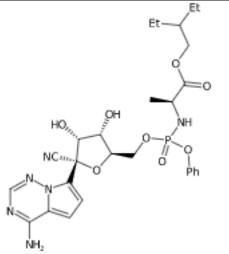	Nucleoside analogue	Yes (a suggested prodrug treatment for ebola and COVID-19).	No inhibition.	-9.5	-7.0
stavudine	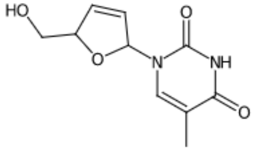	Nucleoside analogue	Yes (reverse transcriptase inhibitor; management of HIV).	No inhibition.	-5.8	-5.7
deferoxamine	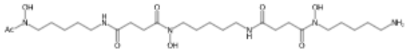	Chelating agent	Yes (treatment of iron overdose, haemachromatosis, aluminium toxicity).	No inhibition.	-6.9	-6.3
auranofin	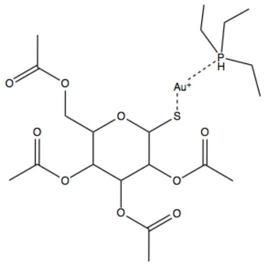	Thiol-reactive	Yes (treatment of rheumatoid arthritis).	Incomplete inhibition, IC_{50} unable to be calculated.	Not modelled.	Not modelled.
γ-ATP	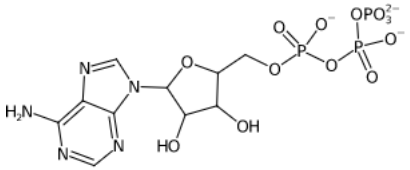	Nucleotide analogue	No.	Some inhibition.	-7.4	-7.2

GS441524	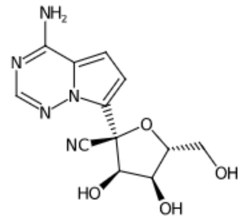	Nucleoside analogue	No (but is the main physiological metabolite of remdesivir).	No inhibition.	-7.1	-6.3
CldU	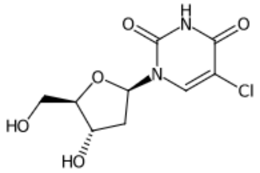	Nucleoside analogue	No.	No inhibition.	-7.0	-6.1
IdU	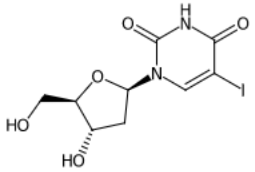	Nucleoside analogue	No.	No inhibition.	-6.6	-6.3
mirin	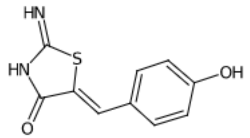	Nuclease inhibitor (Mre11)	No.	No inhibition.	-7.2	-5.7
7-ACA	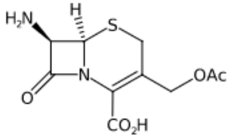	Antibiotic	No (but is a core chemical structure of the cephalosporin antibiotics).	No inhibition.	-6.2	-5.9

olaparib	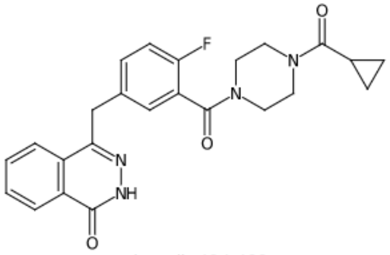	PARP inhibitor	Yes (chemotherapeutic; used in the treatment of homologous recombination defective breast and ovarian cancers).	No inhibition.	-11.4	-9.1
etoposide	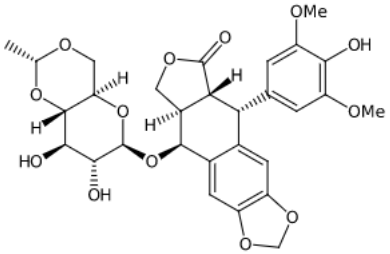	Topoisomerase inhibitor	Yes (chemotherapeutic; broadly used, including testicular cancer, lung cancer, lymphoma).	No inhibition.	-8.4	-7.8
R9-AMK	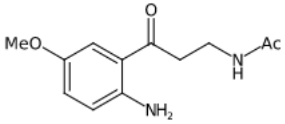	Tryptophan derivative/variant	No.	Some inhibition.	-6.9	-5.5
valproic acid	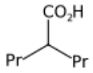	GABAergic compound, HDAC inhibitor	Yes (treatment of epilepsy and bipolar disorder).	Some inhibition.	-5.0	-4.3
camptothecin	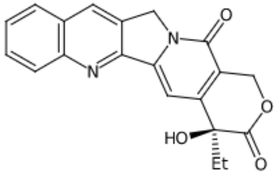	Topoisomerase inhibitor	No (but four camptothecin analogues are approved for cancer chemotherapy).	Incomplete inhibition, IC_{50} unable to be calculated.	-8.0	-8.1

thiram	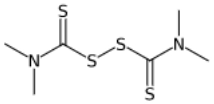	Thiol-reactive	No.	Yes, IC_{50} = 48.2 μM.	-3.7	-3.4
R7 AFMK	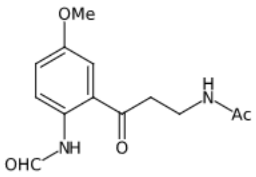	Tryptophan derivative/variant	No.	Some inhibition.	-6.3	-5.5
6-OH-melatonin	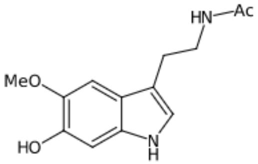	Tryptophan derivative/variant	No.	Some inhibition	-6.2	-5.8
N-ac-serotonin	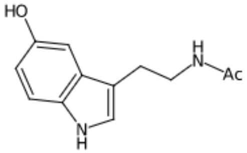	Tryptophan derivative/variant	No.	Some inhibition.	-7.1	-6.0
5FOA	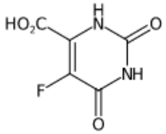	Derivative of pyrimidine precursor	No.	Some inhibition.	-6.2	-5.4

captopril	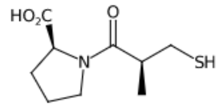	ACE inhibitor	Yes (treatment of hypertension and congestive heart failure).	No inhibition.	-5.2	-4.5
PBIT	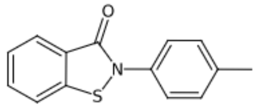	Thiol-reactive	No (variant of ebselen).	Some inhibition.	-8.6	-6.0
disulfiram	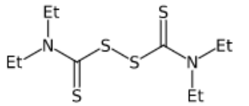	Thiol-reactive	Yes (acetaldehyde inhibitor, management of alcoholism).	Yes, IC_{50} value = 89 µM.	-4.0	-3.9
IPA3	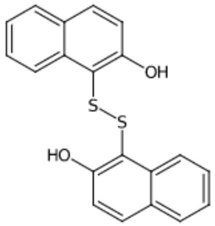	Kinase inhibitor	No.	Some inhibition.	-9.5	-7.6
6-thio-guanine	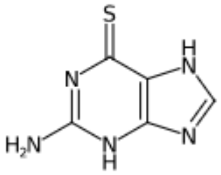	Purine base analogue	Yes (chemotherapeutic; used in the treatment of acute myeloid leukaemia, acute lymphocytic leukaemia, chronic myeloid leukaemia).	No inhibition.	-5.7	-5.0

8-OH 5-nitroquinolone	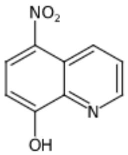	Quinolone	No.	No inhibition.	-7.2	-5.5
NSC9537	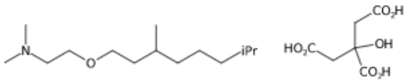	Kinase inhibitor	No.	Some inhibition.	-5.0	-4.7
APE inhibitor III	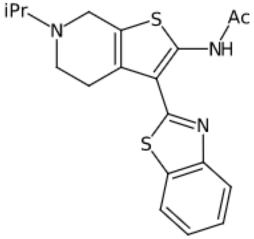	Nuclease inhibitor (APE1)	No.	Some inhibition.	Not modelled.	Not modelled.
azidothymidine	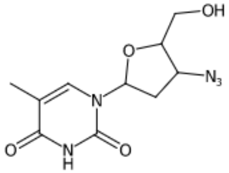	Nucleoside analogue	Yes (reverse transcriptase inhibitor; management of HIV).	No inhibition.	-6.6	-5.8
azidothymidine-triphosphate	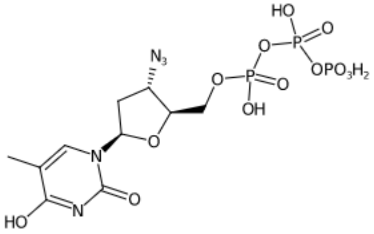	Nucleoside analogue	No (but is the main physiological metabolite of azidothymidine).	No inhibition.	-7.9	-6.7

stavudine-triphosphate	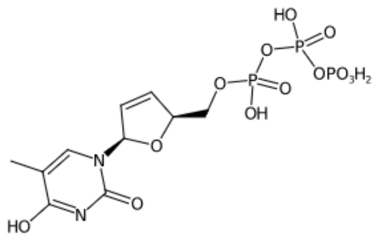	Nucleoside analogue	No (but is the main physiological metabolite of stavudine).	No inhibition.	-7.8	-6.8
lamivudine-triphosphate	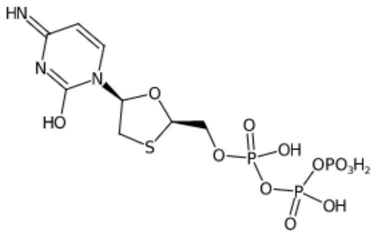	Nucleoside analogue	No (but is the main physiological metabolite of lamivudine).	Some inhibition.	-7.7	-6.3
dATP	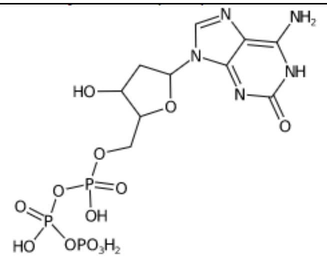	Nucleotide	No (but is physiologically abundant).	No inhibition.	-8.4	-7.4
dTTP	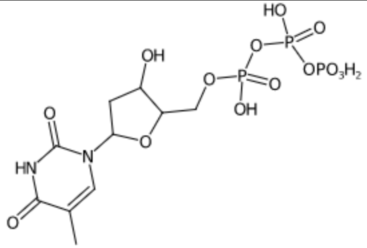	Nucleotide	No (but is physiologically abundant).	No inhibition.	-7.7	-7.8

thymidine	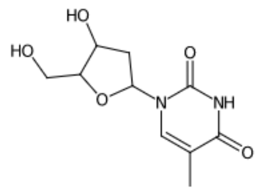	Nucleoside	No (but is physiologically abundant).	No inhibition.	-7.5	-6.2
norfloxacin	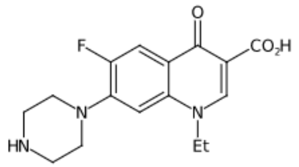	Antibiotic	Yes (fluoroquinolone antibiotic; broad antibacterial usage).	No inhibition.	-9.0	-6.5
nalidixic acid	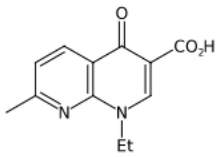	Antibiotic	Yes (quinolone antibiotic; broad antibacterial usage).	No inhibition.	-7.7	-6.3
AZ1353160 (AZ-A1)	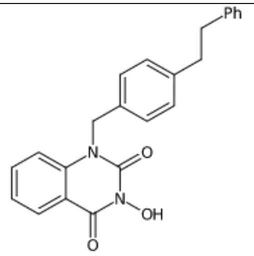	Nuclease inhibitor (FEN1)	No.	Incomplete inhibition, IC_{50} unable to be calculated.	-10.3	-8.5
AZ13623940 (AZ-B1)	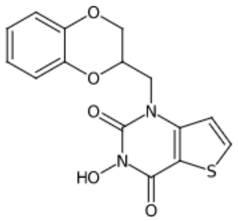	Nuclease inhibitor (FEN1)	No.	Incomplete inhibition, IC_{50} unable to be calculated.	-8.4	-6.7

hycanthone	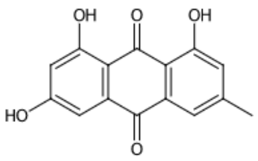	Acetylcholinesterase inhibitor/DNA intercalator	Yes (antiparasitic, used to treat schistosomiasis).	No inhibition.	-9.5	-7.2
adenine	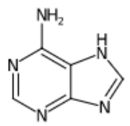	Nucleobase	No (but is physiologically abundant).	No inhibition.	-5.5	-4.7
kynuramine	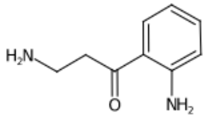	Tryptophan derivative/variant	No.	No inhibition.	-6.1	-4.4
serotonin-HCl	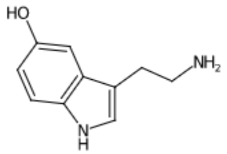	Tryptophan derivative/variant	No (but is physiologically abundant).	Some inhibition.	-6.6	-5.1
A-1	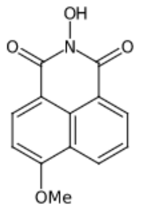	*N*-hydroxyimide	No.	IC_{50} value unable to be determined.	-9.1	-6.8

A-2	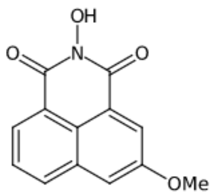	*N*-hydroxyimide	No.	Yes, IC_{50} value = 20.7 μM.	Not modelled.	Not modelled.
H2	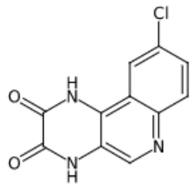	2,3-dihydroxypyrazine scaffold	No.	No inhibition.	-8.2	-6.7
A-3	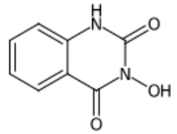	*N*-hydroxyimide	No.	IC_{50} value unable to be determined.	-7.4	-5.8
A-4	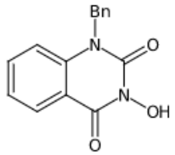	*N*-hydroxyimide	No.	Yes, IC_{50} value = 94.1 μM.	-8.7	-7.1
A-5	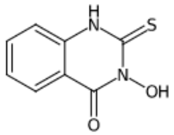	*N*-hydroxyimide	No.	IC_{50} value unable to be determined.	-7.1	-5.3

B-1	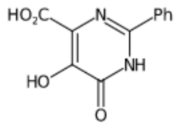	Hydroxypyrimidone	No.	Yes, IC_{50} value = 32.2 μM.	-8.1	-6.5
raltegravir	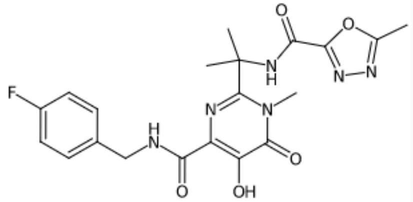	Hydroxypyrimidone	Yes (integrase inhibitor, used in the treatment of HIV).	Yes, IC_{50} value = 24.2 μM.	-8.9	-7.7

Supplementary Table 2. List of compounds tested for *in vitro* inhibition on nsp14-nsp10 nuclease activity.
Compound structures, functional groupings, and FDA approval status are shown alongside *in vitro* inhibition activity and AutoDock Vina scores of the highest-affinity binding modes for each compound. See Fig. 5, Fig. 6, Suppl. Fig. 4, and Suppl. Fig. 5 for relevant data.

Supplementary Table 3

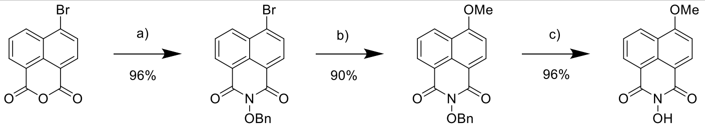

1 2 3

Scheme A-1: *a) $NH_2OBn.HCl$, pyridine, reflux, 2 h; b) NaOMe, $CuSO_4$, MeOH, reflux, 12 h; c) Pd/C, H_2, DMA, RT, 4 h.*

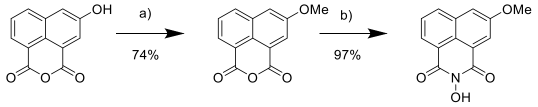

4 5

Scheme A-2: *a) SO_4Me_2, K_2CO_3, acetone, reflux 3 h; b) $NH_2OH.HCl$, pyridine, reflux, 1 h.*

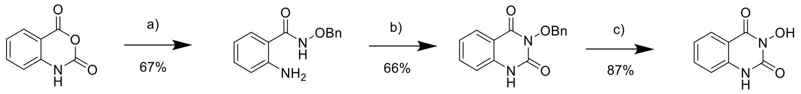

6 7 8

Scheme A-3: *a) $NH_2OBn.HCl$, TEA, THF, reflux, 3 h; b) triphosgene, THF, 0 °C, 2 h; c) Pd/C, H_2, DMF, RT, 2 h.*

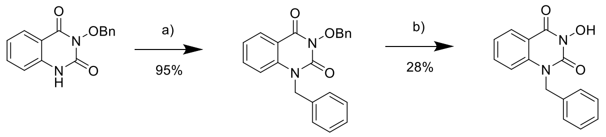

7 9 10

SchemeA-4: *a) BnBr, Cs_2CO_3, DMF, 80 °C, 2 h; b) conc. HBr, H_2O, AcOH, reflux, 1 h.*

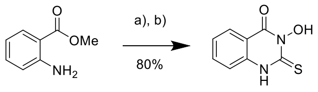

11

Scheme A-5: *a) thiophosgene, H_2O, DCM, RT, 12 h; b) $NH_2OH.HCl$, NaOH, H_2O, $CHCl_3$ RT, 2h.*

Suppl. Table 3: Synthesis scheme for N-hydroxyimide compounds A-1 to A-5

See Suppl. Materials and Methods for detailed synthesis protocols.

Reference

1. Lorenz, R., Bernhart, S.H., Honer Zu Siederdissen, C., Tafer, H., Flamm, C., Stadler, P.F. and Hofacker, I.L. (2011) ViennaRNA Package 2.0. *Algorithms Mol Biol*, **6**, 26.
